# Human Induced Pluripotent Stem Cells Are Targets for Allogeneic and Autologous Natural Killer (NK) Cells and Killing Is Partly Mediated by the Activating NK Receptor DNAM-1

**DOI:** 10.1371/journal.pone.0125544

**Published:** 2015-05-07

**Authors:** Vanessa Kruse, Carina Hamann, Sebastian Monecke, Lukas Cyganek, Leslie Elsner, Daniela Hübscher, Lutz Walter, Katrin Streckfuss-Bömeke, Kaomei Guan, Ralf Dressel

**Affiliations:** 1 Department of Cardiology and Pneumology, University Medical Center Göttingen, Göttingen, Germany; 2 Department of Cellular and Molecular Immunology, University Medical Center Göttingen, Göttingen, Germany; 3 DZHK (German Center for Cardiovascular Research), Partner site Göttingen, Germany; 4 Primate Genetics Laboratory, German Primate Center, Göttingen, Germany; Karolinska Institutet, SWEDEN

## Abstract

Human induced pluripotent stem cells (hiPSCs) could be used to generate autologous cells for therapeutic purposes, which are expected to be tolerated by the recipient. However, iPSC-derived grafts are at risk of giving rise to teratomas in the host, if residuals of tumorigenic cells are not rejected by the recipient. We have analyzed the susceptibility of hiPSC lines to allogeneic and autologous natural killer (NK) cells. IL-2-activated, in contrast to resting NK cells killed hiPSC lines efficiently (P=1.69x10^-39^). Notably, the specific lysis of the individual hiPSC lines by IL-2-activated NK cells was significantly different (P=1.72x10^-6^) and ranged between 46 % and 64 % in ^51^Cr-release assays when compared to K562 cells. The hiPSC lines were killed by both allogeneic and autologous NK cells although autologous NK cells were less efficient (P=8.63x10^-6^). Killing was partly dependent on the activating NK receptor DNAM-1 (P=8.22x10^-7^). The DNAM-1 ligands CD112 and CD155 as well as the NKG2D ligands MICA and MICB were expressed on the hiPSC lines. Low amounts of human leukocyte antigen (HLA) class I proteins, which serve as ligands for inhibitory and activating NK receptors were also detected. Thus, the susceptibility to NK cell killing appears to constitute a common feature of hiPSCs. Therefore, NK cells might reduce the risk of teratoma formation even after autologous transplantations of pluripotent stem cell-derived grafts that contain traces of pluripotent cells.

## Introduction

Pluripotent stem cells hold great promises for regenerative medicine. They might become a source of cells and tissues for new cell replacement therapies, e.g. to treat heart failure or Parkinson’s disease. However, the use of human embryonic stem cells (ESCs) for the development of new transplantation therapies is restricted due to ethical concerns. Induced pluripotent stem cells (iPSCs) obtained by reprogramming of somatic cells by a set of defined pluripotency factors might overcome this problem [1–3]. In addition, iPSCs have the advantage that they can potentially be used not only in allogeneic but also in autologous settings. Autologous iPSC-derived grafts would substantially reduce the risk of immune rejection, but they might on the other hand be at higher risk of teratoma formation than allogeneic transplants if residuals of pluripotent cells remain in the grafts after *in vitro* differentiation into the desired cell type or tissue [4]. This assumption is based on results showing that murine ESCs and iPSCs give rise to teratomas in immunocompetent syngeneic but usually not in immunocompetent allogeneic mice [4–9].

In mice, several immune effector mechanisms appear to be important for the rejection of pluripotent stem cell-derived teratomas in allogeneic hosts, including T cells [10, 11] and the complement system [12]. We have shown previously that murine pluripotent stem cells, including ESCs and iPSCs, are targets for allogeneic and syngeneic NK cells [5, 13]. NK cells can delay teratoma growth after transplantation of pluripotent stem cells although they are not sufficient to suppress teratomas completely, if the stem cells are injected in high enough numbers [4, 13, 14]. Notably, murine NK cells also impaired the growth of human ESCs (hESCs) in a xenograft model [15]. Recently, a porcine iPSC line was reported to be killed by swine leukocyte antigen (SLA)-matched as well as SLA-mismatched NK cells [16]. Notably, these iPSCs failed to form teratomas in SLA-matched hosts although they formed tumors in immunodeficient mice [16]. Thus, NK cells might contribute to rejection of MHC-matched iPSCs also in a large animal-model.

On NK cells, inhibitory and activating receptors are expressed and the balance of activating and inhibitory signals determines their activation against target cells [17]. Inhibitory receptors on human NK cells include killer cell immunoglobulin-like receptors (KIRs), which recognize certain allelic groups of the classical peptide-presenting HLA-A, HLA-B, and HLA-C class I molecules and the CD94-NKG2A receptor, which recognizes the non-classical class I molecule HLA-E that presents preferentially leader peptides derived from other HLA class I molecules. The KIR receptor 2DL4 recognizes HLA-G, a further non-classical class I molecule, which is mainly expressed in the placenta [18] but was reported to be expressed also in hESCs [19]. Lack of HLA class I molecules can trigger the cytotoxic activity of NK cells. The ‘missing self’ concept describes this way of NK cell activation [20]. Activating receptors of human NK cells include natural-killer group 2, member D (NKG2D), which recognize the MHC class I chain-related proteins (MIC)A and MICB and the UL16-binding proteins (ULBP1-6). The activating DNAX accessory molecule (DNAM)-1 recognizes CD155 (the poliovirus receptor) and CD112 (Nectin-2). The activating receptor 2B4 interacts with CD48. Activating KIRs recognize certain allotypes of HLA class I molecules and can therefore play a role in the rejection of allogeneic targets. A further group of activating receptors are the natural cytotoxicity receptors (NCRs) NKp30, NKp44, and NKp46, for which a number of different ligands have been described during the last years although several of them are debated [17]. Many ligands of activating NK receptors such as MICA and MICB are not expressed by normal cells but become induced by cellular or genotoxic stress in virus-infected or malignant cells. The ‘stress-induced self’ concept refers to this way of NK cell activation [21].

Murine pluripotent stem cells lack MHC class I molecules [9, 13, 22, 23] at least at a level detectable by flow cytometry [24]. In mice, MHC class I molecules interact with inhibitory Ly49 receptors on NK cells, a family of receptors that functionally replace KIRs in rodents. Thus, the ‘missing self’ can contribute to the killing of mouse ESCs and iPSCs by NK cells. In addition, we found ligands for the activating NK receptors NKG2D and DNAM-1 on murine pluripotent stem cells and demonstrated that killing of these cells by NK cells depends in part on NKG2D [5, 13].

Human ESCs have been described to express low but in flow cytometry detectable amounts of HLA class I molecules [25–27]. They were also reported to express low amounts of ligands of the activating NK receptor NKp46 [26] and the NKG2D ligands MICA and MICB [28]. Similarly, human iPSCs (hiPSCs) were recently shown to express low amounts of HLA class I molecules [29, 30] and NKp46 ligands [29]. In accord with this phenotype, NK cells can kill hESCs at least at a moderate level [26, 31]. In contrast, the hESC line H9 was reported not to induce degranulation and interferon-γ release in NK cells [32, 33]. However, resting NK cells require stimulation by cytokines to acquire full cytotoxicity against many targets [34] and such stimulation is likely provided by a pro-inflammatory milieu after transplantation. Therefore, we investigated in this study the lysis of hiPSC lines by allogeneic and autologous NK cells activated by low doses of interleukin-2 (IL-2) and analyzed the expression of ligands of activating and inhibitory NK receptors. The results showed that hiPSC were targets for both allogeneic and autologous IL-2-activated NK cells.

## Materials and Methods

### Generation and culture of hiPSC lines

Three hiPSC lines (D1-iPSC4, D2-iPSC1 and D3-iPSC3) used in this study were generated from hair keratinocytes of three healthy donors (donor 1, 2 and 3), respectively. The generation of D1-iPSC4 (also named as Kera4-iPS4) and D2-iPSC1 (also named as Kera2-iPS1) lines was described in our previous study [35]. The D3-iPSC3 line was derived from a 30-year-old healthy male by using the STEMCCA system [36], which is a humanized excisable lentivirus system containing the four reprogramming factors OCT4, SOX2, KLF4, and c-MYC in a single ‘stem cell cassette’ (pHAGE2-EF1aFull-hOct4-F2AhKlf4-IRES-hSox2-P2A-hcMyc-W-loxP). All three hiPSC lines were characterized for their pluripotency within this study as described previously [35]. A further hiPSC line (D6-iPSC2) used for some experiments has been characterized previously and was named FB2-iPS2 [35]. The ethics committee of the University Medical Center Göttingen has approved the study (Az 21/1/11). The participants have given their written informed consent to participate in this study. The ethics committee has approved the consent procedure. The hiPSC lines were expanded on mitomycin C-inactivated mouse embryonic fibroblasts (MEFs) as described previously [35]. To avoid contamination with MEFs, the cells were transferred to Matrigel-coated (BD Biosciences, Heidelberg, Germany) dishes and cultured in MEF-conditioned medium three to four days before being used for experiments. The cell lines were used at passages 31 to 46 (D1-iPSC4), 31 to 44 (D2-iPSC1), 13 to 23 (D3-iPSC3), and 25 to 29 (D6-iPSC2).

### Effector cells and ^51^Cr-release assay

Peripheral blood mononuclear cells (PBMCs) were obtained from healthy donors by centrifugation on Biocoll separating solution (Biochrom, Berlin, Germany) as described previously [34]. NK cells were isolated from PBMCs by magnetic-activated cell sorting (MACS) using negative selection kits (NK cell isolation kit II, Miltenyi Biotec, Bergisch-Gladbach, Germany) and either used directly or cultured for four days with 200 U/ml human IL-2 (Proleukin, Chiron, Amsterdam, Netherlands). Unseparated PBMCs were cultured for four days with 200 U/ml IL-2 to obtain lymphokine-activated killer (LAK) cells. K562 cells served as reference cell line in ^51^Cr-release assays and were cultured as described previously [34]. Target cells were labeled by incubating 1 x 10^6^ cells in 200 μl Dulbecco’s modified Eagle medium (DMEM) containing 100 μl fetal calf serum (FCS) and 50 μCi Na_2_^51^CrO_4_ (CrRA8, Hartmann Analytic, Braunschweig, Germany) for 1 h at 37°C and washed three times with DMEM. Effector cells were added to 5 x 10^3^ ^51^Cr-labeled target cells in triplicates at various ratios in 200 μl DMEM with 10% FCS per well of round-bottomed microtiter plates. In some experiments, monoclonal antibodies (mAbs) were added at a concentration of 10 μg/ml (anti-NKG2D, anti-DNAM-1, anti-ICAM-1 and mouse IgG_1_ as isotype control or anti-HLA class I W6/32 HL [37] and as control the non-binding variant W6/32 HK [38]). Spontaneous release was determined by incubation of target cells in the absence of effector cells. In blocking experiments, the respective mAbs were added also to the wells, which served to determine the spontaneous release. The microtiter plates were centrifuged for 5 min at 40 x g, incubated at 37°C for 4 h, and then centrifuged again. Supernatants and Triton X-100-lysed sediments were separately taken to determine radioactivity in each well using a MicroBeta^2^ counter (PerkinElmer Life Sciences, Köln, Germany). Percentage of specific lysis was calculated by subtracting the spontaneous ^51^Cr release.

### Quantitative polymerase chain reaction (qPCR)

Total RNA was extracted from cell lines, treated with DNase I to avoid contamination with genomic DNA, and used for cDNA synthesis as described previously [24]. The analyzed genes and the primer pairs used are given in S1 Table. Amplification reactions were carried out in 96-well plates in 20 μl reaction volumes with the Power SYBR green PCR master mix (Applied Biosystems, Foster City, USA). The PCR reaction plates were preheated for 2 min at 50°C and for 10 min at 95°C followed by 40 cycles of denaturation (15 s at 95°C) and amplification (1 min at 60°C). All reactions were performed in technical triplicates using an ABI 7500 Real Time PCR System. For the data analysis, the ABI 7000 system SDS software (Applied Biosystems) was used. To determine primer amplification efficiencies, each primer pair was tested in serial cDNA dilutions (1:5, 1:25, 1:125, 1:625) and a standard curve was plotted showing the cycle threshold (ct)-values over the logarithm of cDNA amount to calculate the slope (m) of the standard curve. Primer amplification efficiencies (E) were calculated with the formula E = 10^(-1/m)^. Expression of housekeeping genes (*GAPDH*, *HPRT* and *ACTB*) was determined in parallel to the genes of interest. The internal control gene stability measure M of each housekeeping gene was calculated to select the most appropriate reference gene [39]. *GAPDH* had the smallest M value (M = 0.04) compared to *HPRT* (M = 0.08) and *ACTB* (M = 0.12) and was therefore used to normalize variations in cDNA concentration in different samples. The relative amount of transcripts was expressed as Δct value (ct of the gene of interest minus ct of *GAPDH*). The formula given by Pfaffl and colleagues [40] was used to calculate the mRNA expression difference to a calibrator. PBMCs and K562 cells were used as calibrators of gene expression in the hiPSC lines.

### Flow cytometry

Flow cytometry was performed with a FACSCalibur flow cytometer and CellQuestPro software (BD Biosciences). Cell surface expression of molecules of interest on hiPSCs was tested on propidium iodide negative cells with the mAbs shown in Table 1. In addition, we used recombinant human IgG_1_ Fc chimeric NK receptor proteins to detect ligands of NKG2D (1299-NK), NKp30 (1849-NK), NKp44 (2249-NK), and NKp46 (1850-NK) all purchased from R&D Systems, Wiesbaden, Germany. The hiPSC lines were tested by flow cytometry in parallel to the ^51^Cr-release and CD107a degranulation assays so that the reported results characterize directly the stem cells that were functionally tested. For K562 cells data from additional experiments were included in the analysis. We used 0.5 μg of the mAbs or the recombinant receptors to stain 1 x 10^6^ cells in 100 μl phosphate-buffered saline (PBS). A fluorescein isothiocyanate (FITC)-conjugated goat anti-mouse IgG Ab (155-095-062, Jackson Laboratories, via Dianova, Hamburg, Germany) served as secondary reagent for the mAbs and a FITC-conjugated goat anti-human IgG Ab (109-095-098, Jackson Laboratories) as secondary reagent for the recombinant receptor molecules (1 μl/100 μl PBS). Staining with secondary Abs alone served as controls. NK cells were characterized using mAbs against the NK cell markers indicated. The respective antibodies and isotype controls are listed in Table 1. All stainings were performed at 4°C in the dark.

**Table 1:** Antibodies used in the study.

Antigen	Isotype	Clone	Label^1^	Supplier
2B4 (CD244)	mouse IgG_1_	C1.7	PE	BioLegend, Fell, Germany
CD3	mouse IgG_2a_	HIT3a	FITC	BioLegend
CD16	mouse IgG_1_	3G8	PE/Cy5	BioLegend
CD56	mouse IgG_1_	HCD56	PE	BioLegend
CD56	mouse IgG_1_	HCD56	APC	BioLegend
CD94	mouse IgG_1_	HP-3D9	FITC	Becton Dickinson, Heidelberg, Germany
CD107a (LAMP-1)	mouse IgG_1_	H4A3	FITC	BioLegend
CD112	mouse IgG_1_	TX31	-	BioLegend
CD155	mouse IgG_1_	Skll.4	-	BioLegend
CD158 (KIR2DL1/S1/S3/S5)	mouse IgG_2b_	HP-MA4	PE	BioLegend
CD158b	mouse IgG_2b_	CH-L	PE	Becton Dickinson
CD158b (KIR2DL2/L3, NKAT2)	mouse IgG_2a_	DX27	PE	BioLegend
CD158d (KIR2DL4)	mouse IgG_1_	33	PE	BioLegend
CD158e/k (KIR3DL1/DL2)	mouse IgG_1_	5133	PE	Miltenyi, Bergisch Gladbach, Germany
CD158e1/e2 (KIR)	recombinant human IgG_1_		PE	Miltenyi
CD158i (KIR2DS4)	mouse IgG_1_	JJC11.6	PE	Miltenyi
CD158f (KIR2DL5)	mouse IgG_1_	UP-R1	PE	BioLegend
DNAM-1 (CD226)	mouse IgG_1_	11A8	PE	BioLegend
DNAM-1 (CD226)	mouse IgG_1_	11A8	-	BioLegend
HLA-A, B, C (HLA class I)	mouse IgG_2a_	W6/32 HL	-	hybridoma supernatant (in house production)
HLA-A, B, C (HLA class I) non-binding variant	mouse IgG_2a_	W6/32 HK	-	hybridoma supernatant (in house production)
HLA-DR (HLA class II)	mouse IgG_2a_	L243	-	hybridoma supernatant (in house production)
HLA-E	mouse IgG_1_	3D12	-	BioLegend
ICAM-1 (CD54)	mouse IgG_1_	HA58	-	BioLegend
MICA	mouse IgG_1_	AMO1	-	Bamomab, Gräfelfing, Germany
MICB	mouse IgG_2a_	BMO2	-	Bamomab
NKG2A (CD159a)	mouse IgG_2a_	131411	PE	R&D Systems, Wiesbaden, Germany
NKG2C (CD159c)		REA205	APC	Miltenyi
NKG2D (CD314)	mouse IgG_1_	149810	PE	R&D Systems
NKG2D (CD314)	mouse IgG_1_	149810	-	R&D Systems
NKp30 (CD337)	mouse IgG_1_	P30-15	PE	BioLegend
NKp44 (336)	mouse IgG_1_	P44-8	APC	BioLegend
NKp46 (CD335)	mouse IgG_1_	9E2	PE	BioLegend
ULBP1	mouse IgG_2a_	AUMO2	-	Bamomab
ULBP2	mouse IgG_1_	BUMO1	-	Bamomab
ULBP3	mouse IgG_1_	CUMO3	-	Bamomab
mouse IgG	goat IgG	polyclonal (155-095-062)	FITC	Jackson Laboratories, via Dianova, Hamburg, Germany
human IgG	goat IgG	polyclonal (109-095-098)	FITC	Jackson Laboratories
isotype control	mouse IgG_1_	PPV-06	FITC	Immunotools, Friesoythe, Germany
isotype control	mouse IgG_1_	PPV-06	PE	Immunotools
isotype control	mouse IgG_1_	MOPC-21	PE/Cy5	BioLegend
isotype control	mouse IgG_2a_	MOPC-173	FITC	Immunotools
isotype control	mouse IgG_2a_	MOPC-173	PE	Immunotools
isotype control	mouse IgG_2b_	PLPV219	PE	Immunotools

^1^APC: allophycocyanin, Cy5: Cyanine 5, FITC: fluorescein isothiocyanate, PE: phycoerythrin.

### CD107a degranulation assay

NK cells were cultured for four days with 200 U/ml human IL-2 and then incubated at a ratio of 4:1 with the respective target cells for 2 h at 37°C. During the last 30 min a FITC-conjugated mAb against CD107a or the respective isotype control (mouse IgG_1_, clone PPV 06) was added together with an allophycocyanin (APC)-conjugated anti-CD56 mAb. Moreover, phycoerythrin (PE)-conjugated mAbs against certain NK cell receptors were added (anti-NKG2D, anti-NKG2A, anti-DNAM-1, or a mixture of anti-KIR mAbs). Antibodies were used at a concentration of 10 μg/ml for 4 x 10^5^ NK cells. Afterwards, the cells were washed with ice-cold PBS and analyzed by flow cytometry.

### *KIR* genotyping

The *KIR* genotyping was done as described by Uhrberg et al. [41].

### Statistics

The data are presented as means ± standard error of the mean (SEM). They were analyzed with the WinStat software (R. Fitch Software, Bad Krozingen, Germany). After testing for normal distribution, analysis of variance (ANOVA) was performed for data consisting of more than two groups followed by Student-Newmann-Keuls post hoc test. Two-way-ANOVA was used to analyze the lysis of different target cell lines by cytotoxic effector cells (adjusted for the effector:target ratio). The non-parametric Kruskal-Wallis (H) test was used if the data were not normally distributed. All results reported to be significant after ANOVA were also significant after a non-parametric analysis using the H test. For two group comparisons, Student’s *t*-test or the non-parametric Mann-Whitney (U) test was utilized. Two-sided P-values of <0.05 were considered as statistically significant. Bonferroni-Holm corrections for multiple testing were performed when appropriate.

## Results

### Pluripotency characterization of human iPSC lines

**Fig 1:**
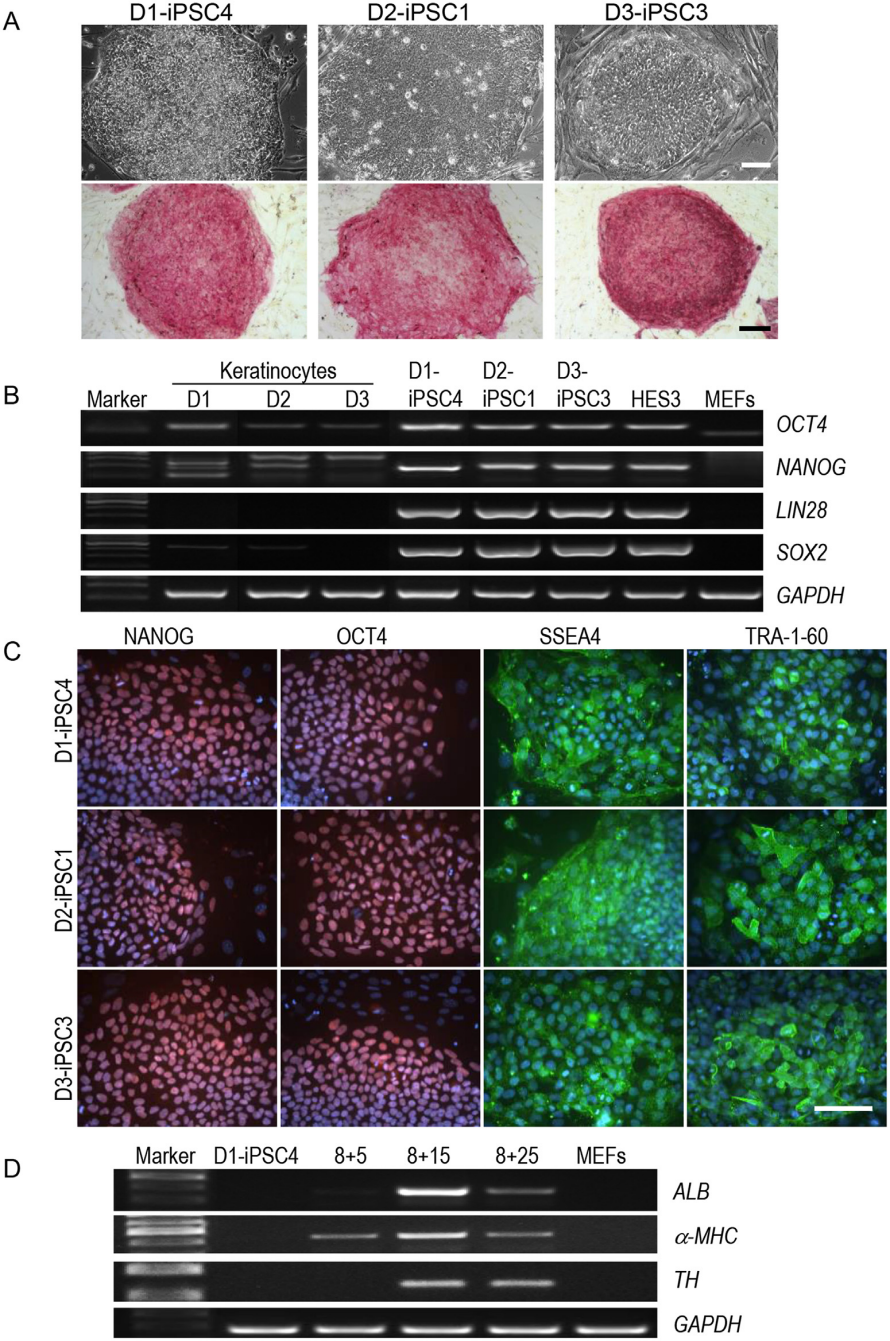
The hiPSC lines showed pluripotent characteristics. (A) The three hiPSC lines with typical morphology for human pluripotent stem cells expressed alkaline phosphatase. Scale bars: 100 μm. (B) The three hiPSC lines expressed endogenous pluripotency markers *OCT4*, *NANOG*, *LIN28*, and *SOX2* at the mRNA level as shown by RT-PCR. *GAPDH* is included as loading control. HES3 cells and MEFs were included as positive and negative control, respectively. (C) Immunofluorescence staining of the hiPSC lines with Abs against pluripotency markers NANOG, OCT4, SSEA4, and TRA1-60. The cells were counterstained with DAPI (blue). Scale bar: 100 μm. (D) Germ layer-specific genes *ALB*, *α-MHC*, and *TH* were expressed in a developmentally controlled manner during EB differentiation. *GAPDH* is included as loading control. Analyses were performed at different stages (days 5, 15, or 25) during differentiation of EBs after plating at day 8 (d8). Marker, 100 bp DNA marker.

The hiPSC lines selected for this study (D1-iPSC4, D2-iPSC1, and D3-iPSC3) were maintained for more than 20 passages without any obvious phenotypic changes. They showed the typical human pluripotent stem cell morphology and were positive for alkaline phosphatase (Fig 1A). RT-PCR analyses showed the activation of endogenous pluripotency genes (*OCT4*, *NANOG*, *LIN28*, and *SOX2*) in all three analyzed hiPSC lines compared to their parental keratinocytes (Fig 1B). In addition, all three iPSC lines were positive for human pluripotent stem cell markers NANOG, OCT4, SSEA4, and TRA-1-60 as demonstrated by immunocytochemical staining (Fig 1C). Thus, the hiPSC lines expressed the typical pluripotency markers. Upon spontaneous differentiation via embryoid body (EB) formation, all three iPSC lines differentiated into derivatives of three embryonic germ layers *in vitro*, as detected by expression of genes specific for endoderm, albumin (*ALB*), for mesoderm, α-myosin heavy chain (*α-MHC*), and for neuroectoderm, tyrosine hydroxylase (*TH*) (Fig 1D). The hiPSC line D6-iPSC2 that was used in some experiments was also maintained for more than 20 passages without any phenotypic changes and has been extensively characterized elsewhere [35].

### Killing of hiPSCs by autologous and allogeneic NK cells

**Fig 2:**
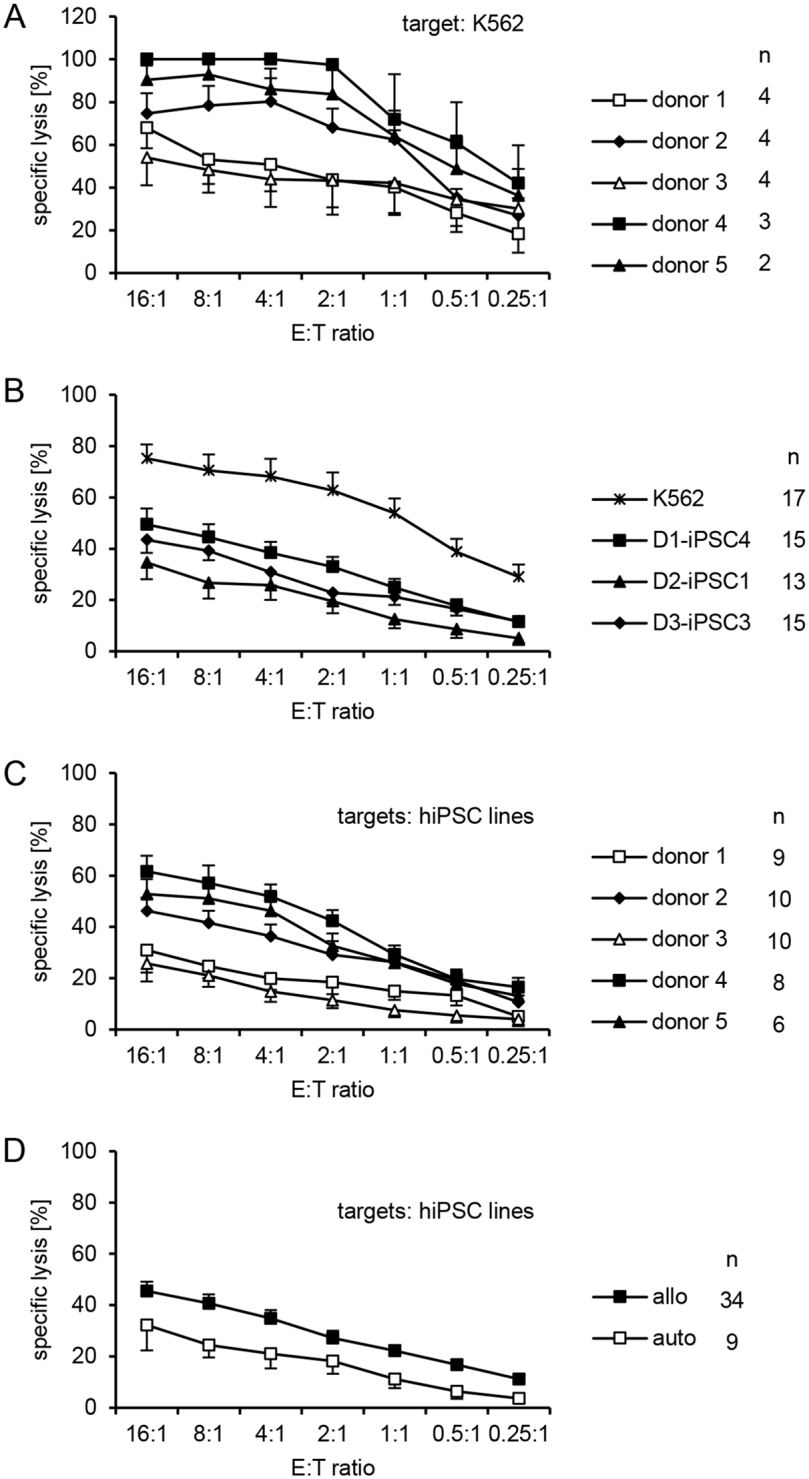
Human iPSC lines were killed by purified and IL-2-activated NK cells of various donors but allogeneic effector cells were more efficient than autologous NK cells. (A) NK cells were stimulated for four days with IL-2 (200 U/ml) and used as effector cells against the reference target cell line K562 in ^51^Cr-release assays. Each individual test was done in triplicates. The means of specific lysis and the standard error of the mean (SEM) at different effector:target (E:T) ratios (16:1 to 0.25:1) are shown to summarize these experiments. The numbers of individual experiments (n) are indicated in the figure. (B) A summary of means of specific lysis and the SEM of K562 and three hiPSC lines by IL-2-activated NK cells from five donors (1 to 5) is shown. (C) A summary of means of specific lysis and the SEM of the three hiPSC lines (D1-iPSC4, D2-iPSC1, D3-iPSC3) by NK cells of five different donors is shown. (D) A summary of means of specific lysis and the SEM of the three hiPSC lines (D1-iPSC4, D2-iPSC1, D3-iPSC3) by allogeneic (allo) and autologous (auto) NK cells is shown.

Three hiPSC lines (D1-iPSC4, D2-iPSC1, and D3-iPSC3) were used as targets for purified IL-2-activated NK cells from the three donors of the keratinocytes (donors 1, 2 and 3) and two further unrelated blood donors (donors 4 and 5) in ^51^Cr-release assays. Thus, each hiPSC line was a target for autologous and allogeneic NK cells obtained from four different unrelated donors and each combination was tested in two to four independent replications (S1 Fig). The NK cell sensitive cell line K562 was always included in the experiments as reference target cell line. We noticed that the NK cells from the five different donors varied in their efficacy to kill K562 cells (Fig 2A) (P = 1.08x10^-8^, 2-way-ANOVA adjusted for E:T ratio). The cytotoxic activity of NK cells of donors 1 and 3 was relatively low and significantly different from the other donors. Therefore, the killing of K562 cells at the highest effector to target ratio (16:1) was set for some evaluations to 100% and the relative lysis of the other target cell lines and at the various effector to target ratios was calculated in every experiment accordingly (S2 Fig, see also right panels in S1 Fig). The relative lysis of K562 by NK cells from the various donors (panel A in S2 Fig) was not significantly different due to this adjustment (P = 0.1690, H test).

The further analysis of these data indicated that the hiPSC lines were killed by NK cells from the five donors although to a lesser degree than K562 cells (Fig 2B and panel B in S2 Fig). Over all experiments, the three hiPSC lines differed significantly from each other in their susceptibility to NK cell-mediated lysis (specific lysis: P = 1.72x10^-6^, 2-way-ANOVA adjusted for E:T ratio, relative lysis: P = 1.27x10^-5^, H test). The D1-iPSC4 cells were most susceptible and the D2-iSPC1 cells most resistant to NK cells. NK cells from the different donors varied also in their activity against the three hiPSC lines (Fig 2C) (specific lysis: P = 7.25x10^-25^, 2-way-ANOVA adjusted for E:T ratio) forming two groups of either relatively inefficient (donors 1 and 3) or relatively efficient killers (donors 2, 4, and 5). This effect remained stable after adjustment for differences in the efficacy to kill K562 cells (panel C in S2 Fig) (relative lysis: P = 8.15x10^-8^, 2-way-ANOVA adjusted for E:T ratio), suggesting that the ability of NK cells from these two donors to kill hiPSCs was particularly low. The hiPSC lines were in general killed by both autologous and allogeneic NK cells, however, allogeneic NK cells were more efficient than autologous NK cells (Fig 2D and panel D in S2 Fig) (specific lysis: P = 8.63x10^-6^, relative lysis: P = 9.98x10^-5^, H test). These differences remained significant even when the results obtained with NK cells of the donors 4 and 5, for which we had no autologous iPSC lines and which killed all hiPSC lines efficiently, were removed from the analysis (data not shown).

Next, we analyzed the results obtained with allogeneic and autologous NK cells separately. The three hiPSC lines differed clearly in their susceptibility to allogeneic NK cells (panel A in S3 Fig) (specific lysis: P = 4.34x10^-7^, relative lysis: P = 2.12x10^-8^, 2-way-ANOVA adjusted for E:T ratio) and D2-iPSC1 cells were most resistant. However, the allogeneic NK cells varied also in their cytotoxic activity against the three hiPSC lines (panel B, left part in S3 Fig) (specific lysis: P = 4.64x10^-15^, 2-way-ANOVA adjusted for E:T ratio). This effect remained again stable after adjustment for differences in efficacy to kill K562 cells (panel B, right part in S3 Fig) (relative lysis: P = 2.12x10^-8^, 2-way-ANOVA adjusted for E:T ratio). The efficacy of killing by autologous NK cells was also different for the three hiPSC lines (panel C in S3 Fig) (specific lysis: P = 0.0024, relative lysis: P = 0.0116, 2-way-ANOVA adjusted for E:T ratio).

**Fig 3:**
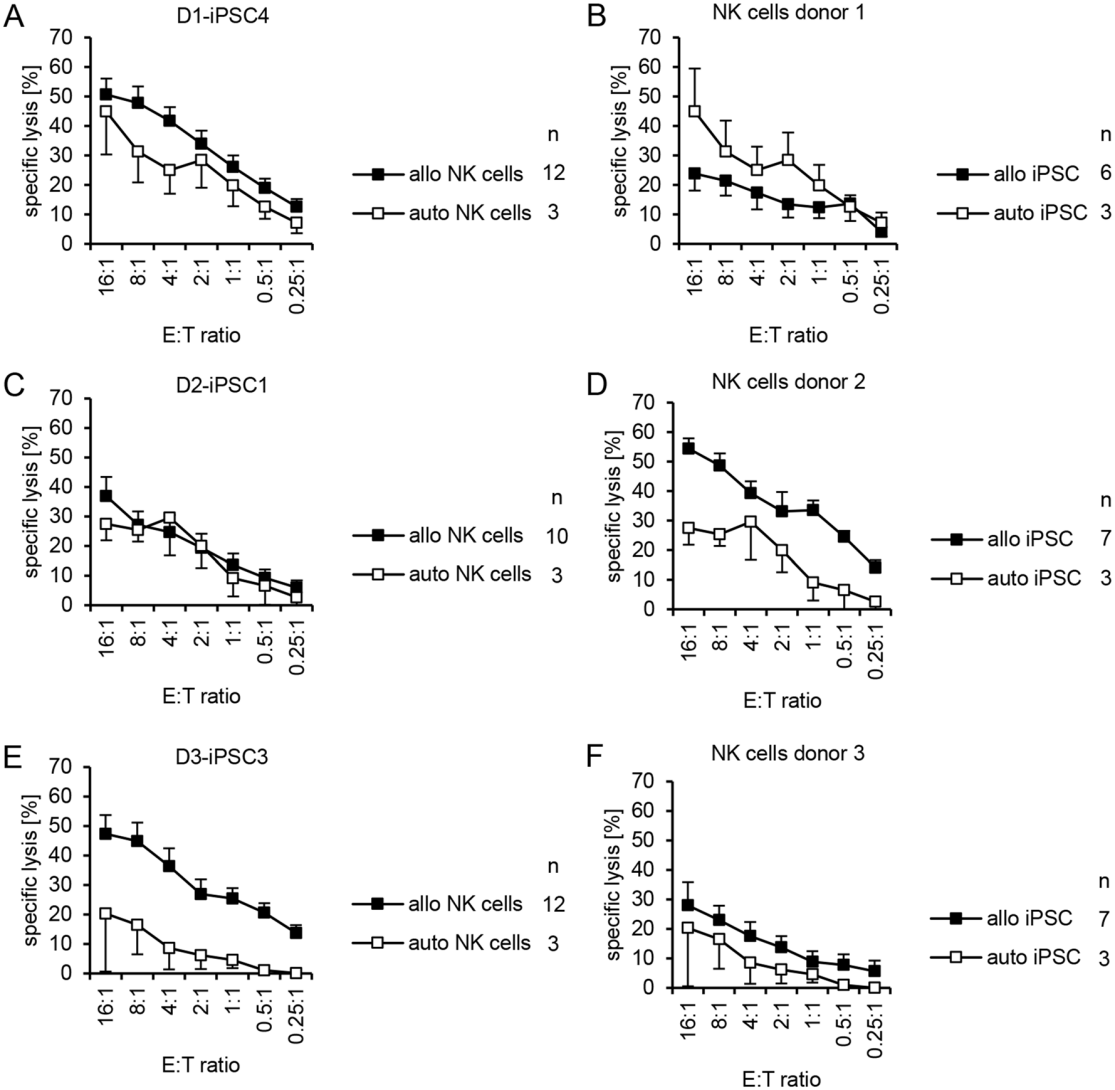
Individual hiPSCs varied in their susceptibility to allogeneic and autologous NK cells and NK cells of individual donors varied in their activity against allogeneic and autologous hiPSCs. (A) A summary of means of specific lysis and the SEM of D1-iPSC4 cells by allogeneic (allo) and autologous (auto) NK cells is shown. The numbers of individual experiments (n) are indicated in the figure. (B) A summary of means of specific lysis and the SEM of the autologous hiPSC line D1-iPSC4 and the two allogeneic hiPSC lines (D2-iPSC1, D3-iPSC3) by NK cells of donor 1 is shown. (C) A summary of means of specific lysis and the SEM of D2-iPSC1 cells by allogeneic (allo) and autologous (auto) NK cells is shown. (D) A summary of means of specific lysis and the SEM of the autologous hiPSC line D2-iPSC1 and the two allogeneic hiPSC lines (D1-iPSC4, D3-iPSC3) by NK cells of donor 2 is shown. (E) A summary of means of specific lysis and the SEM of D3-iPSC3 by allogeneic (allo) and autologous (auto) NK cells is shown. (F) A summary of means of specific lysis and the SEM of the autologous hiPSC line D3-iPSC3 and the two allogeneic hiPSC lines (D1-iPSC4, D2-iPSC1) by NK cells of donor 3 is shown.

The hiPSC line D1-iPSC4 showed a trend towards a higher susceptibility to allogeneic than autologous NK cells (Fig 3A) (specific lysis: P = 0.0683, ANOVA, P = 0.0326, H test) although NK cells of donor 1 surprisingly killed the autologous D1-iPSC4 cells even better than allogeneic hiPSCs (Fig 3B) (specific lysis: P = 0.0345, ANOVA). NK cells of donor 1 almost failed to kill the allogeneic D3-iPSC3 line (panel A in S1 Fig). The D2-iPSC1 cells were killed similarly but at a low level by autologous and allogeneic NK cells (Fig 3C) (specific lysis: P = 0.5688, ANOVA). NK cells of donor 2 killed allogeneic hiPSCs much better than the autologous D2-iPSC1 cells (Fig 3D) (specific lysis: P = 3.80x10^-6^, ANOVA). D2-iPSC1 cells appeared to be generally more resistant to NK cells than the other hiPSC lines (see also Fig 2B and panel B in S2 and panel A in S3 Figs). Line D3-iPSC3 was killed poorly by autologous NK cells in contrast to allogeneic NK cells (Fig 3E) (specific lysis: P = 7.58x10^-6^, ANOVA). However, NK cells of donor 3 showed in general rather low cytotoxic activity although there was a trend towards a more efficient killing of allogeneic hiPSC lines (Fig 3F) (specific lysis: P = 0.0539, ANOVA, P = 0.0145, H test).

To exclude differences in the percentage of NK cells among the effector cells of the different donors after 4 days of stimulation with IL-2, we determined the proportion of cells expressing CD16, CD56, and CD94 and found no difference when tested by ANOVA (data not shown). In parallel to these experiments with purified and IL-2-activated NK cells, we also used unseparated IL-2-activated PBMCs as effector cells and obtained in principle identical results (data not shown).

### Killing of hiPSCs by IL-2-activated allogeneic NK cells compared to resting NK cells

**Fig 4:**
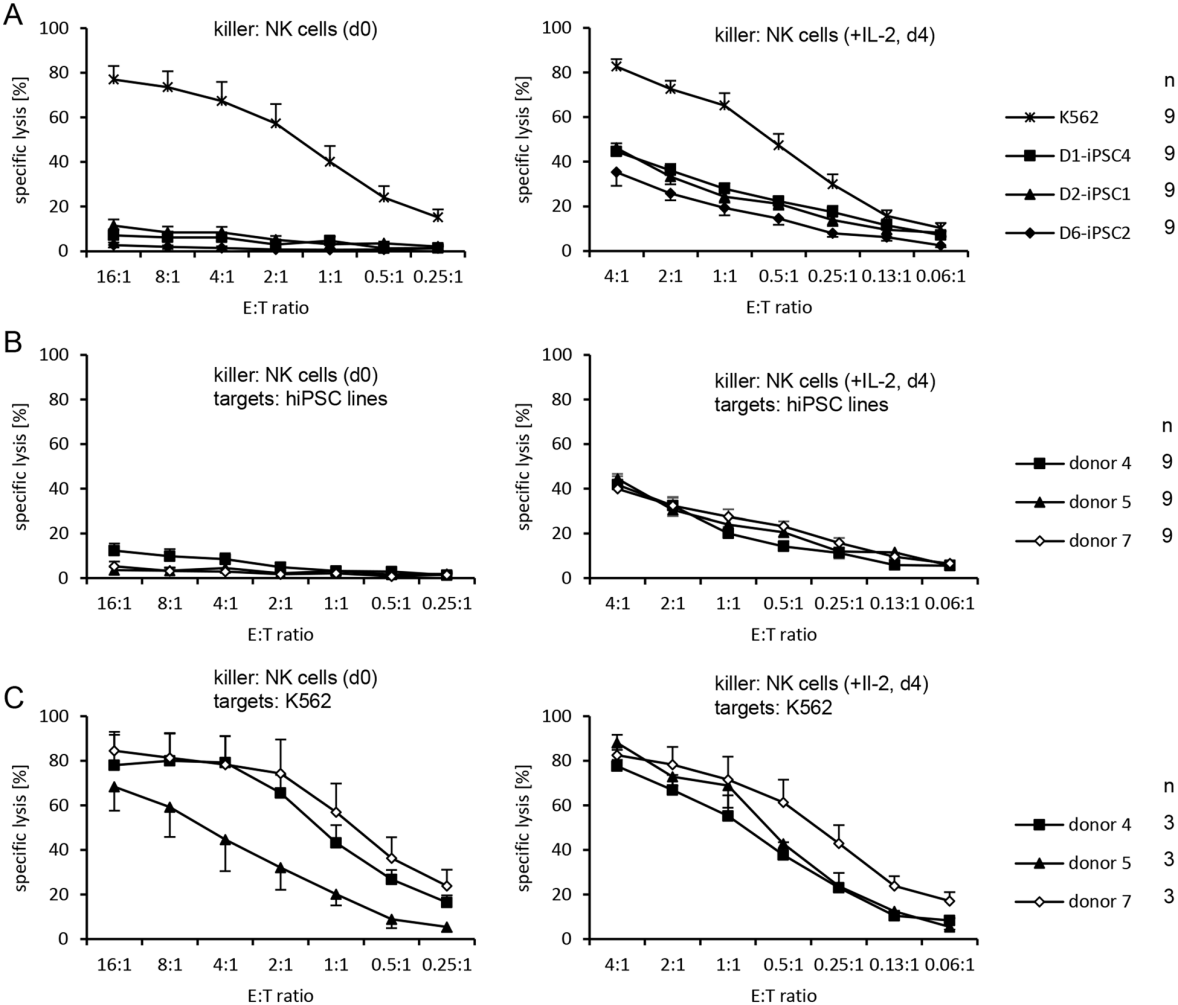
Resting NK cells largely fail to kill hiPSC lines in contrast to K562 cells. (A) A summary of means of specific lysis and the SEM of K562 and three hiPSC lines by freshly isolated resting (day 0, left panel) and IL-2-activated NK cells (day 4, right panel) from the three donors 4, 5, and 7 is shown. The numbers of individual experiments (n) are indicated in the figure. (B) A summary of means of specific lysis and the SEM of the three hiPSC lines (D1-iPSC4, D2-iPSC1, D6-iPSC2) by resting and IL-2-activated NK cells of three NK cell donors is shown. (C) A summary of means of specific lysis and the SEM of K562 cells by resting and IL-2-activated NK cells of three NK cell donors is shown. The E:T ratio ranges from 16:1 to 0.25:1 for resting NK cells and from 4:1 to 0.06:1 for IL-2-activated NK cells.

In the next set of experiments, we compared the specific lysis of hiPSC lines by resting and IL-2-activated allogeneic NK cells. Three hiPSC lines (D1-iPSC4, D2-iPSC1, and D6-iPSC2) were used as targets for freshly purified resting (day 0) and IL-2-activated NK cells (day 4) from three unrelated blood donors (donors 4, 5, and 7) in ^51^Cr-release assays. Thus, each hiPSC line was a target for resting and IL-2-activated NK cells obtained from three different allogeneic donors and each combination was tested in three independent replications (S4 Fig). The hiPSC lines were again efficiently killed by IL-2-activated NK cells but hardly by resting NK cells (E:T ratios 4:1 to 0.25:1, P = 1.69x10^-39^, H test). K562 cells, in contrast, were killed by both resting and activated NK cells (Fig 4A and 4C). At a very low level of killing, the hiPSC lines differed in their susceptibility to resting NK cells and D6-iPSC2 cells were most resistant (Fig 4A, left panel) (P = 1.17x10^-5^, H test). This cell line was also slightly more resistant to IL-2-activated NK cells than the other two hiPSC lines (Fig 4A, right panel) (P = 3.50x10^-7^, 2-way- ANOVA adjusted for E:T ratio). Resting NK cells from the three donors varied slightly in their (low) activity against the hiPSC lines and NK cells of donor 4 were more active than those of donors 5 and 7 (Fig 4B, left panel) (P = 0.0143, H test). The activity of the IL-2-activated NK cells against the hiPSC lines was similar in this set of experiments (Fig 4B, right panel). However, killing of K562 cells by resting (P = 1.58x10^-5^) and IL-2-activated NK cells (P = 0.0003, 2-way-ANOVA adjusted for E:T ratio) differed slightly for the three donors (Fig 4C).

The NK cells used in these experiments were characterized in more detail by a set of NK cell markers (S5 Fig). Fresh NK cells of the three donors varied significantly in the proportion of NKG2C^+^, KIR^+^, NKp46^+^, and NKp30^+^ NK cells (P<0.05, ANOVA, after Bonferroni-Holm correction). The stimulation with IL-2 for 4 days led to an increase of CD94^+^ NK cells (P<0.05, ANOVA, after Bonferroni-Holm correction). The donors were also genotyped for their KIR loci (S2 Table) and their NK cells were tested for reactivity with a panel of anti-KIR mAbs (S6 Fig).

### Characterization of NK cell receptors involved in killing of hiPSC lines

**Fig 5:**
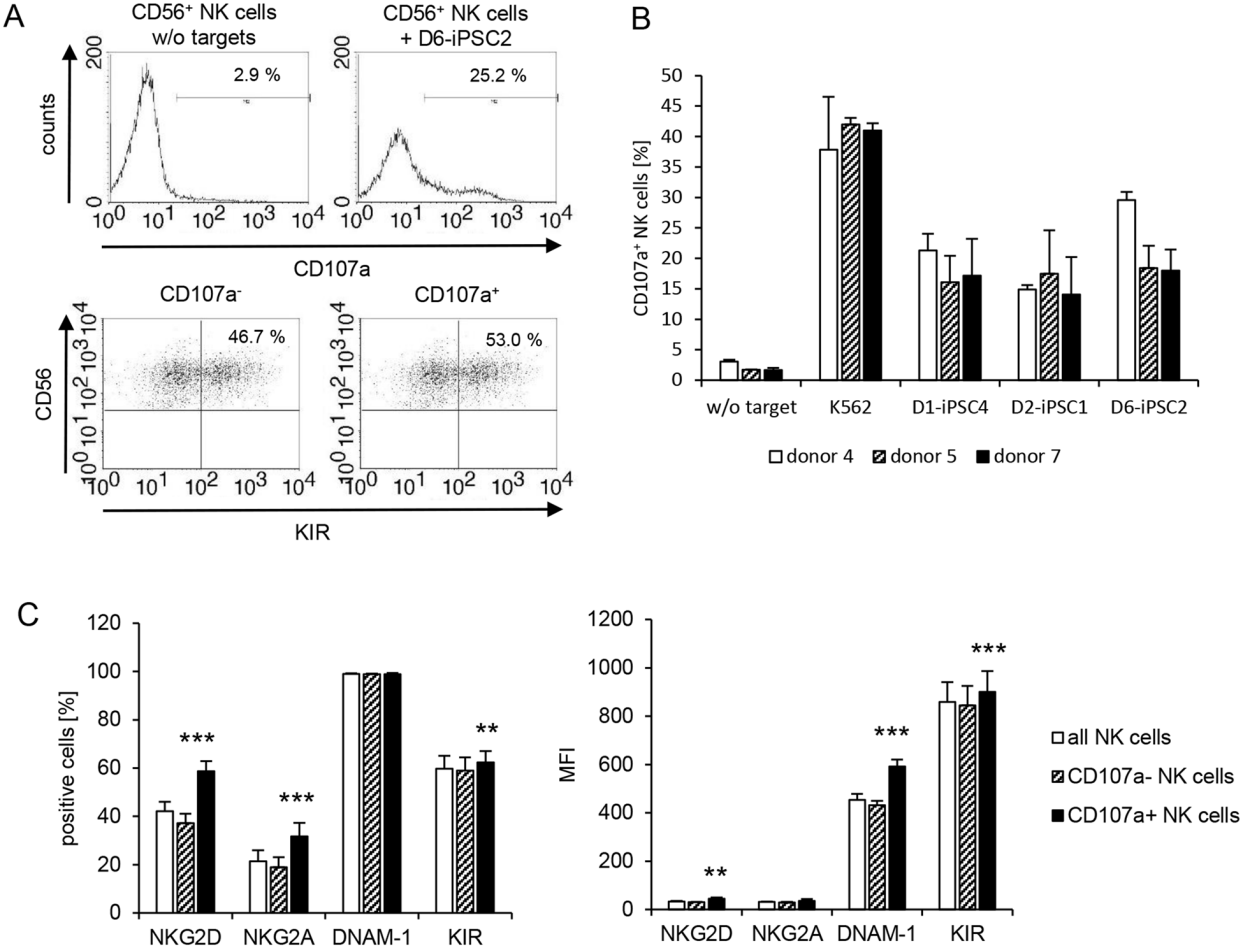
NK cells degranulating in response to hiPSC lines are enriched for several NK cell receptors. (A) The degranulation of IL-2-activated NK cells of donor 7 in response to D6-iPSC2 cells is shown. Without contact to hiPSCs only 2.9% of the NK cells expressed the degranulation marker CD107a. After co-culture with target cells for 2 h 25.2% of the CD56^+^ NK cells expressed CD107a at the plasma membrane. 46.7% of the CD107a^-^ NK cells were KIR positive. Among the CD107a^+^ NK cells 53.0% were KIR positive. The KIR staining was performed with a mixture of all anti-KIR mAbs indicated in Table 1 to cover all KIR molecules. (B) A summary of means and the SEM of CD107a^+^ NK cells of donors 4, 5, and 7 after exposure to three hiPSC lines (D1-iPSC4, D2-iPSC1, D6-iPSC2) and K562 cells is shown (n = 3). (C) In the left panel a summary of means and the SEM of NKG2D^+^, NKG2A^+^, DNAM-1^+^, and KIR^+^ cells among all NK cells exposed to the hiPSCs as well as CD107a^-^ and CD107a^+^ NK cells is shown. In the right panel a summary of means and the SEM of the MFI of NKG2D, NKG2A, DNAM-1, and KIR on all NK cells exposed to the hiPSCs as well as CD107a^-^ and CD107a^+^ NK cells is shown. Significant differences between CD107a^-^ and CD107a^+^ NK cells are indicated (n = 26, *** P<0.001, ** P<0.01, *t*-test after Bonferroni-Holm correction).

To determine which NK cells might be involved in the killing of hiPSC lines, we performed CD107a degranulation assays in parallel to the ^51^Cr-release assays with IL-2-activated NK cells. NK cells became positive for the degranulation marker CD107a upon exposure to hiPSC lines (Fig 5A). However, in these assays we did not observe significant differences between the three hiPSC lines or the NK cells of the three blood donors (Fig 5B). We determined the expression of NKG2D, NKG2A, DNAM-1 and KIR on all NK cells, on CD107a^-^ NK cells, and on CD107a^+^ NK cells exposed to the hiPSC lines as illustrated in Fig 5A for KIR. The CD107a^+^ NK cells were enriched for NKG2D^+^, NKG2A^+^, and slightly for KIR^+^ NK cells (Fig 5C, left panel). Moreover, the CD107a^+^ NK cells expressed more DNAM-1 and slightly more NKG2D and KIR molecules as determined by increased mean fluorescence intensities (MFI) values (Fig 5C, right panel). When the NK cells were exposed to K562 cells, the NK cell receptor expression pattern of CD107a^+^ NK cells was very similar (S7 Fig). Thus, we observed an enrichment of NK cells expressing the activating receptor NKG2D, the inhibitory receptor NKG2A and the KIR family, which contains inhibitory and activating members. For those parameters, which were significantly different between CD107a^-^ and CD107a^+^ NK cells exposed to hiPSCs, we calculated the difference and performed further analyses. The enrichment of NKG2D^+^, NKG2A^+^, and KIR^+^ NK cells in the CD107a^+^ NK cell population was different between the donors but not between NK cells exposed to the three hiPSC lines (panels A, C, and E in S8 Fig). The increase of DNAM-1 and KIR but not NKG2D expression intensity on CD107a^+^ NK cells (panels B, D, and F in S8 Fig) was also different between the donors. None of these parameters was significantly affected by the hiPSC line to which the NK cells were exposed. The individual results for each donor and each target cell line are shown in S9 Fig. In summary, the composition of the NK cell population reacting against hiPSC lines appeared to depend more on the NK cell donors than on the hiPSC lines. However, an overrepresentation of NK cells expressing specific receptors among the CD107a^+^ NK cells is of course not necessarily indicating that the respective receptor contributes to killing of the targets.

**Fig 6:**
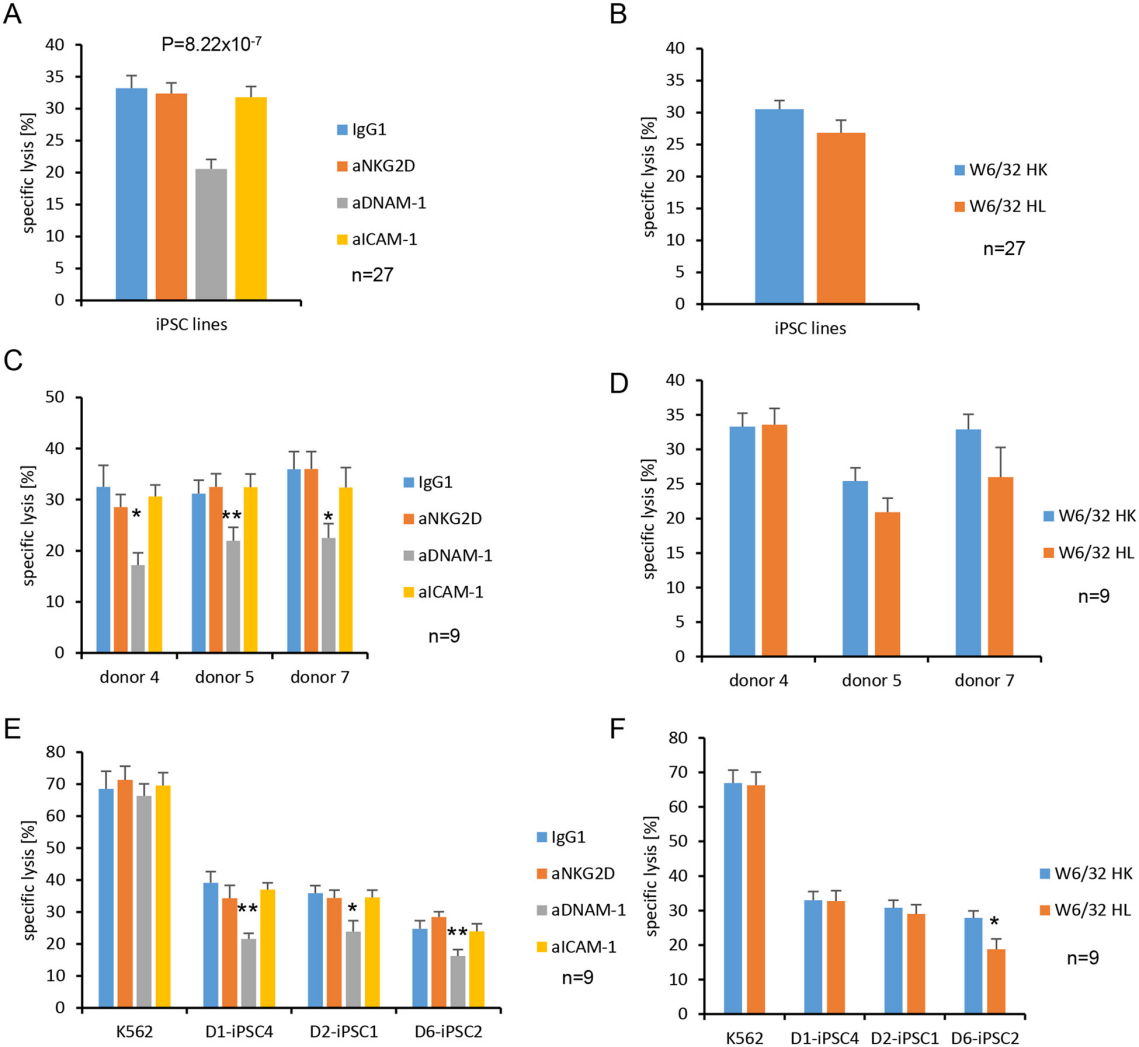
Killing of hiPSC lines is in general dependent on DNAM-1 and for D6-iPSC2 cells also on HLA class I molecules. (A) A summary of means of specific lysis and the SEM of three hiPSC lines (D1-iPSC4, D2-iPSC1, D6-iPSC2) by IL-2-activated NK cells from the donors 4, 5, and 7 in ^51^Cr-release assays is shown after incubation with an isotype control (IgG_1_) or blocking mAbs against NKG2D (aNKG2D), DNAM-1 (aDNAM-1), or ICAM-1 (aICAM-1) at a concentration of 10 μg/ml. The significance of differences was calculated by ANOVA and is indicated in the figure. The numbers of individual experiments (n) are indicated in the figure. (B) A summary of means of specific lysis and the SEM of three hiPSC lines (D1-iPSC4, D2-iPSC1, D6-iPSC2) by IL-2-activated NK cells from the donors 4, 5 and 7 is shown after incubation with the W6/32 HL mAb that binds to HLA class I molecules or as control the non-binding variant W6/32 HK at a concentration of 10 μg/ml. (C, D) The data for the three hiPSC lines are shown grouped according to the three NK cell donors. (E, F) The data are shown grouped according to the three hiPSC lines and K562 cells. Significant differences were calculated by 2-way-ANOVA adjusted either for the NK cell donors or the hiPSC lines and are indicated in the figure (** P<0.01, *P<0.05).

To further clarify which NK cell receptors were indeed important for the killing of hiPSC lines, we performed inhibition experiments in the ^51^Cr-release assays with IL-2-activated NK cells. In general, killing of hiPSCs was partly blocked by an anti-DNAM-1 mAb but not by anti-NKG2D or anti-ICAM-1 mAbs (Fig 6A). The anti-NKG2D mAb was functional since it efficiently blocked killing of MICA expressing L cells [34] (data not shown). To analyze KIR-dependent killing, we used the anti-HLA class I mAb W6/32 HL in comparison to the non-binding variant mAb W6/32 HK that was used as control. Overall, the killing of the hiPSCs was thereby not altered (Fig 6B). A separate analysis for the three NK cell donors indicated that killing of hiPSCs was DNAM-1-dependent for all donors (Fig 6C) and all hiPSC lines (Fig 6E). However, it was not significantly altered by the anti-HLA class I mAb W6/32 HL for any donor (Fig 6D). This mAb blocked partly killing of the D6-iPSC2 line (Fig 6F). An individual analysis for each donor and each hiPSC line (S10 Fig) revealed that the killing of D6-iPSC2 cells by NK cells of donor 7 was significantly inhibited by the anti-HLA class I mAb W6/32 HL (panel F in S10 Fig). The killing of D6-iPSC2 cells by NK cells of donor 5 appeared also to be reduced but only at borderline significance (panel D in S10 Fig) (P = 0.09, *t*-test). This does not prove but suggests that this cell line was killed partly via activating KIRs by NK cells of donor 7 and donor 5, which might have recognized specific HLA class I molecules expressed by this cell line. More individual NK cell reactions against hiPSC lines could exist. NK cells of donor 4, e. g., appeared to kill D1-iPSC4 cells partly in an NKG2D-dependent manner (panel A in S10 Fig) although only at borderline significance (P = 0.09, *t*-test). In summary, DNAM-1 was the most important NK cell receptor for killing of hiPSC lines but on an individual level other receptor ligand pairs appeared to contribute.

### Expression of ligands of activating and inhibitory NK cell receptors in hiPSCs

**Fig 7:**
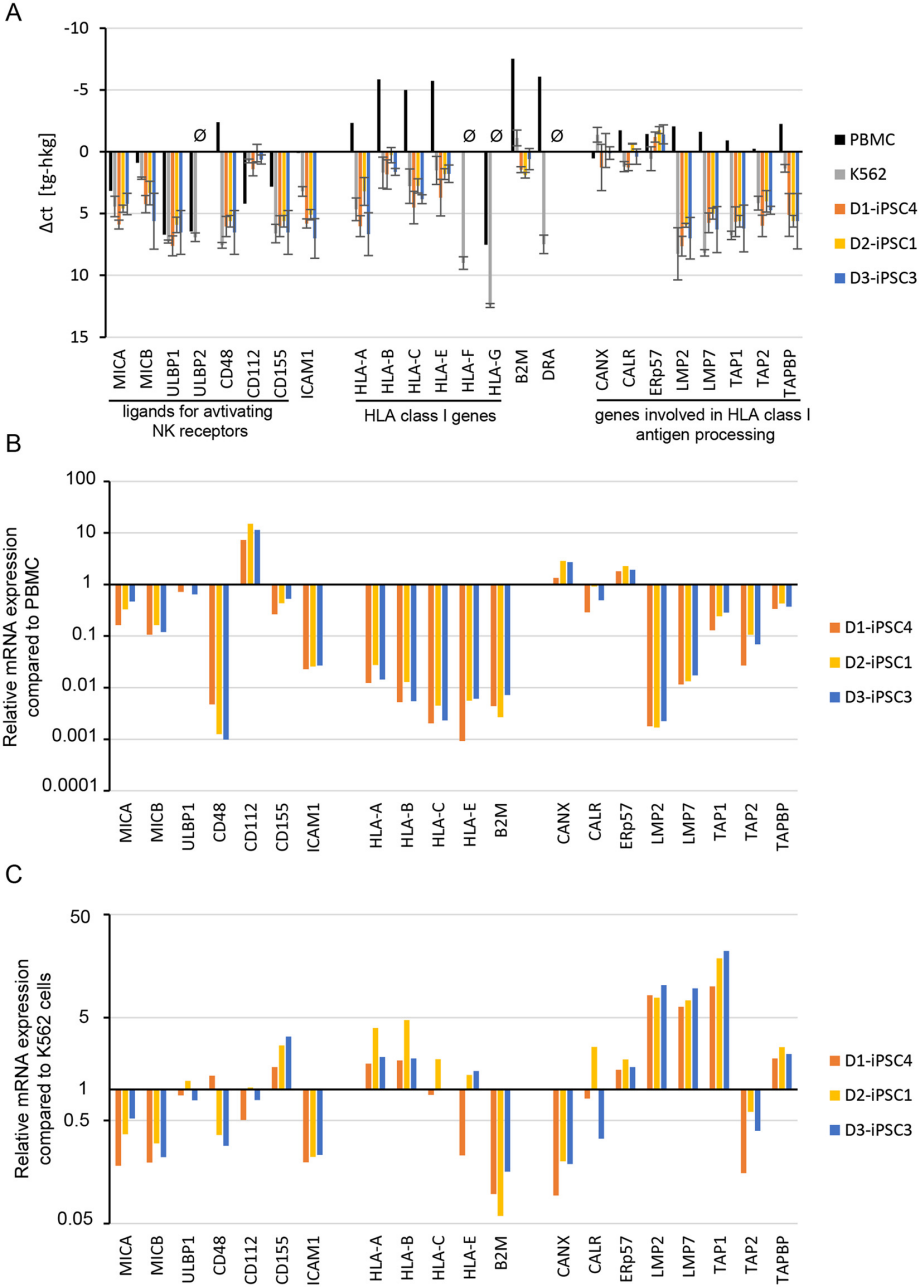
Gene expression analysis of hiPSC lines indicated mostly low mRNA expression of activating and inhibitory NK receptor ligands. (A) The expression of the indicated genes was tested by qPCR in PBMCs, K562 cells and three hiPSC lines (D1-iPSC4, D2-iPSC1, and D3-iPSC3). For the hiPSC lines and K562 cells means ± SEM of Δct values (ct target gene [tg] minus ct housekeeping gene [hkg]) of three biological replicates are shown. Negative values indicated a higher expression of the target gene than the housekeeping gene. Therefore, an inverted scale is shown. At the left side, genes encoding for ligands of activating NK receptors and *ICAM1* are shown. In the middle part classical and non-classical HLA class I genes, *B2M*, and the HLA class II gene *DRA* are shown. In the right part, genes involved in antigen processing in the HLA class I pathway are grouped. Genes not expressed in the iPSC lines (ct > 30) are marked by Ø. (B) The gene expression in the iPSC lines is shown as relative expression compared to PBMCs. (C) The gene expression in the hiPSC lines is shown as relative expression compared to K562 cells.

We then analyzed the gene expression of ligands for activating and inhibitory NK cell receptors in three hiPSC lines by qPCR. Important activating NK receptors are NKG2D, DNAM-1, and 2B4. We analyzed the mRNA expression of the NKG2D ligands *MICA*, *MICB*, *ULBP1*, *ULBP2*, the DNAM-1 ligands *CD112* (*PVRL2*), *CD155* (*PVR*), and the 2B4 ligand *CD48* (Fig 7A). The highest expression levels were found for *CD112*, *MICB*, and *MICA*. *ULPB2* mRNA was not detected in the hiPSC lines analyzed (ct>30). *ICAM1* encodes an adhesion molecule that has been described to be important for killing of mouse ESCs by NK cells [42] and low amounts of this mRNA were detected in the hiPSC lines (Fig 7A). The expression of classical (*HLA-A*, *HLA-B*, *HLA-C*) and non-classical (*HLA-E*, *HLA-F*, *HLA-G*) HLA class I genes was tested together with *B2M*, encoding the non-polymorphic β2-microglobulin that associates with the HLA-class-I α-chain, and the HLA-class-II gene *DRA* (Fig 7A). HLA-A, HLA-B, HLA-C, HLA-E, and HLA-G function as ligands for inhibitory NK receptors. *HLA-B*, *HLA-E*, and *HLA-C* mRNAs were more abundant in the hiPSC lines than *HLA-A* mRNA. *HLA-F*, *HLA-G*, and *HLA-DR* (*DRA*) mRNAs were not detected (ct>30). Since antigen processing is required to obtain normal expression levels of classical class I molecules, we tested the expression of important components of the antigen processing machinery (Fig 7A). The genes of the chaperones calnexin (*CANX*), calreticulin (*CALR*), and Erp57 (*PDIA3*) were strongly expressed in the hiPSC lines, in contrast to the ‘transporter associated with antigen processing’ genes *TAP1*, *TAP2*, and the TAP-binding protein (*TAPBP)* gene as well as genes encoding the specific subunits of the immunoproteasome *LMP2* (*PSMB9*) and *LMP7* (*PSMB8*). The *HLA-A*, *HLA-B*, *HLA-C*, *HLA-E*, *B2M*, *TAP1*, *TAP2*, and *TAPBP* mRNAs were much less abundant in hiPSCs than in PBMCs (Fig 7B), suggesting that the expression of TAP1, TAP2 and TAPBP (Tapasin) might limit the HLA-class-I expression in hiPSC cells. When the gene expression pattern was compared to K562 cells, a higher expression of the *CD155* gene but lower levels of *MICA*, *MICB* and *ICAM1* mRNAs in the hiPSC lines were observed (Fig 7C).

**Fig 8:**
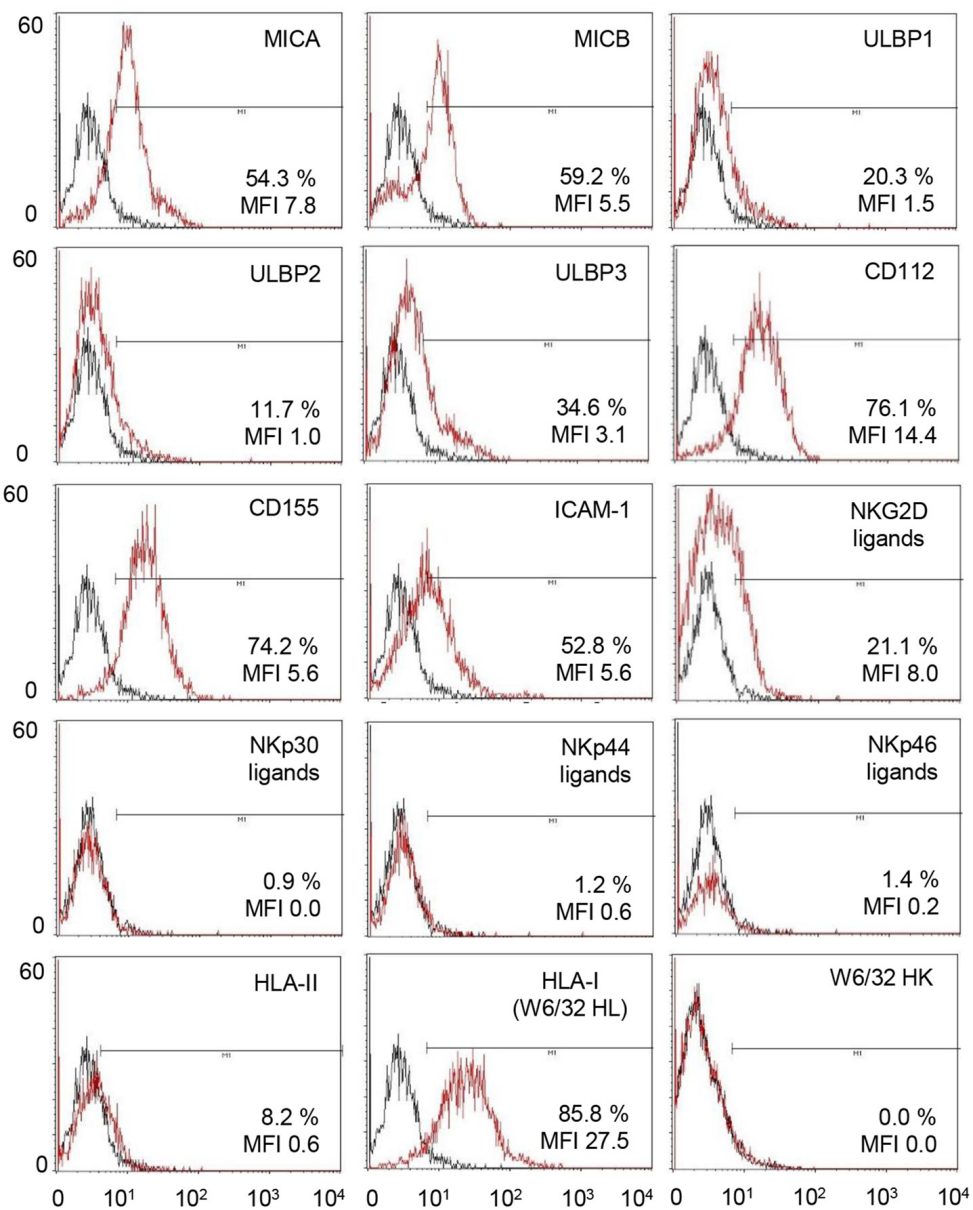
Flow cytometric analysis of ligands for activating and inhibitory NK receptors on D1-iPSC4 cells. Representative histograms of propidium iodide negative D1-iPSC4 cells are shown after staining with mAbs for the indicated molecules or with recombinant receptor molecules (NKG2D, NKp30, NKp44, and NKp46) for the respective ligands. Staining with the respective primary reagent is shown in red and with the secondary Ab only in black. The percentages of specifically stained cells (using the marker shown) and the specific MFI were calculated and are given in the figure.

**Fig 9:**
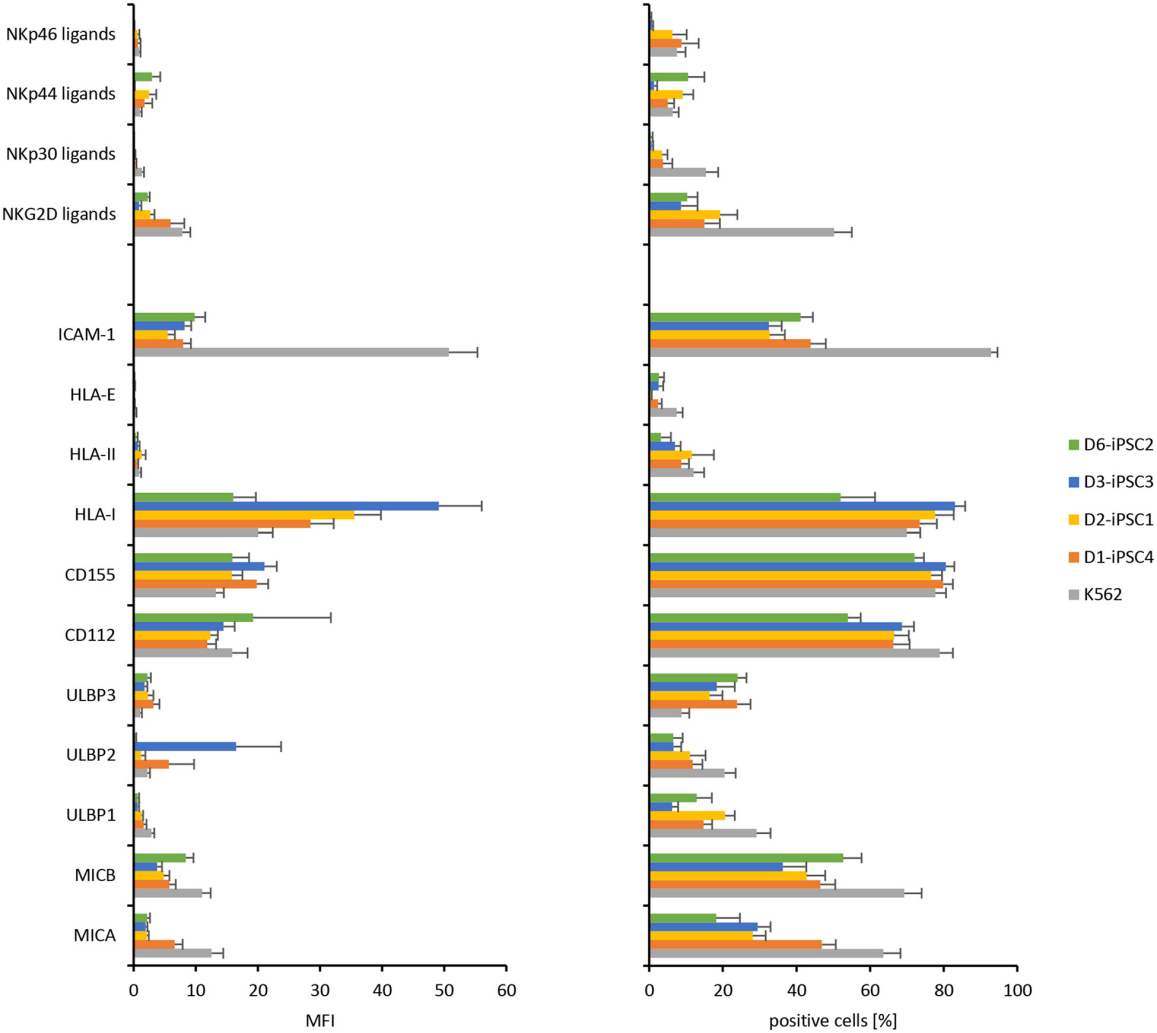
Flow cytometric analysis of ligands for activating and inhibitory NK receptors indicated presence of both classes of molecules on hiPSCs. The expression of the molecules shown in the lower part of the figure was tested in flow cytometry using mAbs and the ligands indicated in the upper part were stained by the recombinant receptor molecules. K562 cells served as control. Shown are means + SEM of the specific MFI (left panel) and the percentage of positive cells (right panel) of K562 (n = 34), D1-iPSC4 (n = 21), D2-iPSC1 (n = 20), D3-iPSC3 (n = 12), and D6-iPSC2 cells (n = 9).

MICA, MICB, CD112, CD155, HLA class I, and ICAM-1 proteins were readily detected on the plasma membrane of the hiPSC lines by flow cytometry as illustrated in Fig 8 for D1-iPSC4 cells. Thus, the hiPSC lines expressed ligands for activating and inhibitory NK receptors as summarized in Fig 9. The CD112 and CD155 and the HLA class I molecules were the most abundant NK receptor ligands on the hiPSC lines. Nonetheless, HLA class I expression intensity was less than 5% compared to PBMCs (data not shown). For the ULBPs and NCR ligands (NKp30, NKp44, NKp46) a complex expression pattern at very low levels was found. When the hiPSC lines and K562 cells were compared with respect to the MFI of the cell surface molecules, K562 cells showed a higher expression of NKG2D ligands (detected with the recombinant receptor molecule and specifically MICA, MICB, ULBP1 detected with mAbs), NKp30 ligands, ICAM-1, and HLA-E (p<0.05, H test after Bonferroni-Holm correction). They expressed less classical HLA class I, ULBP2, and ULBP3 molecules (Fig 9, left panel) (p<0.05, H test after Bonferroni-Holm correction). In accord with these results, a higher proportion of K562 cells than hiPSCs was positive for NKG2D ligands, MICA, MICB, ULBP1, ULBP2, NKP30 ligands, ICAM-1, HLA-E, and CD112 in flow cytometry (p<0.05, H test after Bonferroni-Holm correction). Fewer K562 than hiPSC cells expressed ULBP3 (Fig 9, right panel) (p<0.05, H test after Bonferroni-Holm correction). Overall, this expression pattern could contribute to the higher susceptibility of K562 cells to NK cells compared to the hiPSC cell lines. Comparing the hiPSC lines with each other indicated differences in the expression intensity of MICA (D1-iPSC4 > D2-iPSC1, D3-iPSC3, D6-iPSC2) and HLA class I molecules (D2-iPSC1, D3-iPSC3 > D1-iPSC4, D6-iPSC2) (Fig 9, left panel) (p<0.05 H test after Bonferroni-Holm correction). For MICA also the proportion of positive cells was higher for D1-iPSC4 than the other hiPSC lines, whereas less D3-iPSC3 cells expressed ULBP1 (Fig 9, right panel) (p<0.05, H test after Bonferroni-Holm correction). The consistently higher expression of MICA on D1-iPSC4 cells than on the other two hiPSC cell lines might contribute to the higher susceptibility of this hiPSC line to NK cells at least from some donors.

## Discussion

Recently, the first results of a phase I clinical trial have been reported, in which the transplantation of hESC-derived cells was tested, showing that pluripotent stem cells are on the way to clinical application [43]. Retinal pigment epithelial cells (5 x 10^4^) differentiated from hESCs *in vitro* were injected into a submacular site of two patients with Stargardt’s macular dystrophy or age-related macular degeneration. The clinical results suggested efficacy of these allogeneic transplantations. No signs of hyperproliferation, tumorigenicity, ectopic tissue formation or rejection were observed within the first four months after transplantation. However, the eye is an immune-privileged site so that the information obtained during this trial on the immunogenicity of hESC-derived grafts and the risk of rejection in humans is limited.

Human iPSCs are an alternative source of stem cells to obtain differentiated cells and tissues for transplantations. In contrast to hESCs, hiPSCs can potentially be used in an autologous setting, which reduces in principal the risk of immune rejection. Notably, an unexpected immunogenicity of murine iPSCs in syngeneic hosts has been reported [44] but was contradicted recently by other studies [45–48]. However, currently little is known on the immune response to hiPSCs although the question is highly relevant for the application of hiPSCs in regenerative medicine [49, 50]. Of course, pluripotent stem cells will not be transplanted directly but only after *in vitro* differentiation into the desired cell type or tissue. Nonetheless, it is of interest to determine their susceptibility to immune rejection since they potentially can contaminate stem cell-derived grafts in trace amounts despite efforts to eliminate them quantitatively. Depending on the cell differentiation strategies applied [51], remaining pluripotent cells might confer a residual risk of teratoma formation [52]. This risk would likely be higher in autologous than in allogeneic transplantations [4].

In this study, we have shown that hiPSC lines were susceptible to killing by IL-2-activated NK cells whereas they were largely resistant to freshly isolated resting NK cells. This result is in agreement with a previous report on killing of a hiPSC line by IL-2-activated NK cells [53]. In our experiments, the NK cells killed the hiPSC lines less efficiently than the common NK target cell line K562. This result is in line with our finding that K562 cells expressed more ligands for the activating NK receptor NKG2D than the hiPSC lines. We have shown previously that murine pluripotent stem cells, including iPSCs, were killed by IL-2-activated mouse NK cells with similar efficacy or even better than the common murine NK target cell line YAC-1 [13]. Although, it is difficult to compare K562 and YAC-1 target cells directly, this result might suggest that human iPSCs are less sensitive to NK cells than mouse iPSCs. This could be relevant for the interpretation and extrapolation of results coming from preclinical mouse experiments. Notably, the hiPSCs expressed low amounts of HLA class I molecules as hESCs do [25–27]. The similar phenotype was reported for a few other hiPSC lines [29, 30]. Thus, human pluripotent stem cells appear to engage inhibitory receptors on NK cells better than mouse pluripotent stem cells, which largely lack MHC class I molecules [13, 24].

The killing of all hiPSC lines by NK cells from all donors was partly dependent on DNAM-1 as shown by mAb blocking experiments. Moreover CD107a^+^ NK cells degranulating in response to hiPSC lines expressed more DNAM-1 than the CD107a^-^ NK cells. In addition, other NK receptor ligand interactions might play a role. Interestingly, the cell line D2-iPSC1, which was most resistant to IL-2-activated NK cells (Fig 2B) had the highest HLA class I expression intensity (Fig 8) suggesting an effect of inhibitory KIRs (see Table 2 for a summary of key features of the individual hiPSC lines studied). To further analyze the contribution of KIRs to killing of hiPSCs, we blocked HLA class I molecules on the target cells. In general, this did not alter the lysis significantly. However, the mAb W6/32 was reported to elicit antibody-dependent cellular cytotoxicity of human NK cells [54], which is mediated via CD16 and could have masked effects of blocking the interaction with inhibitory KIRs. Notably, in the specific combination of NK cells of donors 5 and 7 and the D6-iPSC2 target cells, respectively, the blocking of HLA class I molecules reduced lysis suggesting that activating KIRs might have contributed to the killing of this hiPSC line by these NK cells. These NK cell donors had an AB KIR genotype encoding several activating KIRs in contrast to donor 4 who carried an AA genotype lacking activating KIRs with exception of KIR2DS4, which was even not expressed on the NK cells of this donor (S2 Table, S6 Fig). In other combinations, e. g. NK cells of donor 4 and D1-iPSC4 targets, NKG2D-mediated effects appeared to have contributed to killing. We did not observe a dependency of killing on ICAM-1. In summary, the killing of the hiPSC lines was mediated by a NK cell receptor used by all NK cells, i.e. DNAM-1, but other receptors, such as activating KIRs and NKG2D, might have been used in addition in individual NK cell target combinations.

**Table 2:** Summary of features of the hiPSC lines tested in this study.

Feature		D1-iPSC4	D2-iPSC1	D3-iPSC3	D6-iPSC2
**NK receptor ligand expression**	HLA class I	√^1^	√	↑^2^	√
	MICA	↑	√	√	√
	MICB	√	√	√	↑
	CD112	√	√	√	√
	CD155	√	√	√	√
**killing by NK cells**	resting	(+)	(+)	nt^3^	-
	IL-2-activated, allogeneic	++	+	++	+
	IL-2-activated, autologous	++	+	(+)	nt
**dependency of killing on NK receptors**	DNAM-1	+	+	nt	+
	NKG2D	(+) donor 4	-	nt	-
	HLA class I/KIR	-	-	nt	+ donors 5, 7

^1^√: expressed at the plasma membrane.

^2^↑: stronger expressed at plasma membrane in this hiPSC line than on the other hiPSC lines with respect to both MFI and percentage of positive cells.

^3^nt: not tested.

Consistently with these data, all hiPSC lines expressed the DNAM-1 ligands CD112 and CD155. Nonetheless, the three different hiPSC lines, which we investigated in the first set of experiments, varied in their susceptibility to NK cells. The hiPSC lines also varied in the expression of some ligands of NK cell receptors and particularly the NKG2D ligand MICA. The higher expression of MICA on D1-iPSC4 cells than on the other hiPSC lines might have contributed to the higher susceptibility of this hiPSC line to NK cells. However, in the second set of experiments only NK cells from donor 4 appeared to use the NKG2D pathway to kill this hiPSC line. Killing of mouse ESCs and iPSCs by NK cells is known to depend in part on NKG2D [5, 13, 14, 42]. In general, the expression pattern of ligands for activating NK receptors on hiPSCs was very similar to mouse iPSCs, which also expressed NKG2D and DNAM-1 ligands in contrast to 2B4 and NKp40 ligands [13]. So far, we do not know whether the variation among the hiPSC lines is determined by the genetic differences among the cell lines or represents a clonal variability that can occur also among hiPSC lines from the same donor. In accordance with our results, Suárez-Álvarez and colleagues found the NKG2D ligands MICA and MICB by flow cytometry on a hESC line (Shef-1). Moreover, they reported a hiPSC line (MSUH-002) expressing *MICA* and *MICB* but no *ULBP1*, *ULBP2*, and *ULPB3* transcripts [28]. The NKG2D ligands MICA, MICB, ULBP1, ULPB2, and ULBP3 have been shown to be absent on the H9 hESC line [33]. We found a complex expression pattern of NCR ligands (NKp30, NKp44, and NKp46) at very low levels on the hiPSC lines. Chen and colleagues investigated also NKp30, NKp44, and NKp46 ligands on hiPSC lines and found low levels of NKp44 ligands but very little or none of the others [29]. This suggests that NCR ligands are not abundant on hiPSCs but at low levels, variations among cell lines appear to exist.

In our hiPSC lines, *HLA-B*, *HLA-E*, and *HLA-C* mRNAs were more abundant than *HLA-A*. *HLA-F* and *HLA-G* transcripts were not detected. At the plasma membrane, we found low amounts of classical HLA class I molecules but hardly any HLA-E. In the hESC line Shef-1, low levels of *HLA-A* and *HLA-B* but no *HLA-E*, *HLA-F*, and *HLA-G* transcripts were detected [28]. The hiPSC line MSUH-002 was reported to express low amounts of *HLA-B*, *HLA-C*, and *HLA-E* but no *HLA-A* and *HLA-G* mRNAs [28]. Lower levels of transcripts of all classical and non-classical class I genes were recently found in five hiPSC lines compared to their parental cells [29]. In this study, classical HLA class I molecules but hardly any HLA-E and HLA-G were found by flow cytometry on the hiPSC lines [29]. The same pattern has been described for H9 hESCs [33]. Interestingly, *HLA-A* mRNA was less abundant than *HLA-B* and *HLA-C* mRNA in our hiPSC lines and shown to be absent in the MSUH-002 hiPSC line suggesting that the *HLA-A* gene might indeed be consistently repressed in hiPSCs [28].

We investigated also the expression of genes encoding proteins involved in antigen processing in our hiPSC lines. The chaperone genes *CANX*, *CALR*, and *Erp57* were strongly expressed, in contrast to *TAP1*, *TAP2*, and *TAPBP* genes as well as genes encoding the specific subunits of the immunoproteasome *LMP2* and *LMP7*. In the hESC line Shef-1 and the hiPSC line MSUH-002 similarly no or very low expression of the *TAP1* and *TAP2* genes were found [28]. Thus, human pluripotent stem cells appear to lack largely the mRNAs for the transporter proteins, which shuttle peptides from the cytosol to the endoplasmic reticulum and are required for HLA class I plasma membrane expression. This might contribute to the low HLA class I expression on human pluripotent stem cells. Interestingly, an epigenetic H3K9me3 modification was reported to repress *TAPBP* expression, encoding the TAP-binding protein or tapasin, in the MSUH-002 hiPSC line whereas an active H3K4me3 mark was found on the *TAP2* gene only in parental fibroblasts [28]. In addition, decreasing levels of NFκB1 and RelA proteins during reprogramming of hiPSCs can contribute to the low expression of HLA class I molecules in hiPSCs [30]. For the genes encoding chaperones (*CANX*, *CALR*, and *Erp57*) and the specific subunits of the immunoproteasome (*LMP2* and *LMP7*), more cell line specific variations might exist since *CALR* mRNA was not found in Shef-1 hESCs whereas *LMP7* in contrast to *LMP2* mRNA was present [28].

Notably, the hiPSC cell lines investigated in our study were killed not only by allogeneic but also by autologous NK cells, although less efficiently. During their development NK cells undergo a ‘licensing’ or ‘education’ ensuring inhibition of NK cells by self-ligands or NK cell unresponsiveness [55, 56]. Despite the relatively low expression of HLA class I molecules on the hiPSCs lines, NK cell inhibition appeared to be still more efficient by self than non-self HLA class I molecules. However, the individual pattern of susceptibility or resistance against autologous and allogeneic NK cells was surprisingly complex when the individual hiPSC lines were compared. The fact that also NK cells from different donors varied in their efficacy to kill the hiPSC lines contributes to this complexity. Notably, this variance remained stable even after normalization of the results for killing of the reference target cell line K562. Thus, individuals might vary in their ability to reject autologous as well as allogeneic hiPSCs putting them potentially at different risks of tumor formation after transplantation of stem cell-derived grafts that contain residuals of pluripotent cells. A broader set of HLA-typed hiPSC cell lines in addition to KIR-typed NK cell donors will be required to clarify the potential role of KIR-mediated inhibitory and activating effects on hiPSC lines.

Currently, the tumorigenicity of pluripotent stem cells is seen as a major obstacle for stem cell-based therapies [52]. Another hurdle is the immunogenicity of pluripotent stem cells and their derivatives [57–59]. NK cells might be an interesting player in the immune response after transplantation of stem cell-derived grafts since they could primarily target residual pluripotent and tumorigenic cells and spare differentiated cells, which are expected to up-regulate HLA class I molecules during *in vitro* differentiation and therefore inhibit NK cells. NK cells would presumably require activation to target residual pluripotent cells. However, NK cell activation might occur, if the transplantation procedure is associated transiently with an inflammatory response.

## Conclusions

We have shown that both allogeneic and autologous IL-2-activated NK cells can kill hiPSCs and killing was mediated partly by DNAM-1. The activity of NK cells might reduce the risk of teratoma formation after transplantation of pluripotent stem cell-derived grafts that contain traces of pluripotent cells. Since autologous hiPSCs were killed, this could occur even after transplantation of autologous hiPSC-derived grafts. However, variation of NK cell activity against hiPSCs and variation of susceptibility of hiPSCs against NK cells might modify the threshold of undifferentiated hiPSCs that can be tolerated in a graft for a specific recipient.

## Supporting Information

S1 Fig Human iPSC lines were used as target cells for purified and IL-2-activated NK cells of either various allogeneic or autologous donors in ^51^Cr-release assays. The reference target cell line K562 was included in every experiment. Each individual test was done in triplicates. The means of specific lysis and the standard error of the mean (SEM) at different effector:target (E:T) ratios (16:1 to 0.25:1) are shown to summarize these experiments (left panels). In addition, the killing of the reference K562 cells at the highest effector to target ratio (16:1) was set to 100% in each individual experiment and the relative lysis of the other target cell lines and at the various effector to target ratios was calculated accordingly (right panels). The relative lysis is not shown for NK cells of donor 5 since the specific lysis of K562 cells was 100% leading to an identity of specific and relative lysis. The results are grouped with respect to the NK cell donors, i.e. (A) donor 1, (B) donor 2, (C) donor 3, (D) donor 4, and (E) donor 5. In panels A, B, and C, the respective autologous hiPSC line is indicated by open symbols. Allogeneic hiPSC target cell lines are indicated by closed symbols. The numbers of individual experiments (n) are indicated in the figure.
(PDF)

S2 Fig Human iPSC lines were killed by purified and IL-2-activated NK cells of various donors but allogeneic effector cells were more efficient than autologous NK cells. The same data set as in Fig 2 is shown but now the killing of K562 cells at the highest effector to target ratio (16:1) was set to 100% in each individual experiment and the relative lysis of the other target cell lines and at the various effector to target ratios was calculated accordingly. The numbers of individual experiments (n) are indicated in the figure. (A) NK cells from five donors were stimulated for four days with IL-2 (200 U/ml) and used as effector cells against the reference target cell line K562 in ^51^Cr-release assays. Each individual test was done in triplicates. The means of relative lysis and the SEM at E:T ratios 16:1 to 0.25:1 are shown to summarize these experiments. (B) A summary of means of relative lysis and the SEM of K562 and three hiPSC lines by IL-2-activated NK cells from five donors (1 to 5) is shown. (C) A summary of means of relative lysis and the SEM of the three hiPSC lines (D1-iPSC4, D2-iPSC1, D3-iPSC3) by IL-2-activated NK cells of five different donors is shown. (D) A summary of means of relative lysis and the SEM of the three hiPSC lines (D1-iPSC4, D2-iPSC1, D3-iPSC3) by IL-2-activated allogeneic (allo) and autologous (auto) NK cells is shown.
(PDF)

S3 Fig Human iPSC lines were killed by purified and IL-2-activated allogeneic or autologous NK cells of various donors but with different efficacy. (A) A summary of means of specific lysis (left panels) and relative lysis (adjusted to killing of K562 cells, right panels) and the SEM of three hiPSC lines by allogeneic IL-2-activated NK cells from four donors (donors 1 to 5) is shown. The numbers of individual experiments (n) are indicated in the figure. (B) A summary of means of specific lysis (left panel) and relative lysis (right panel) and the SEM of allogeneic hiPSC lines (D1-iPSC4, D2-iPSC1, D3-iPSC3) by NK cells of five different donors is shown. (C) A summary of means of specific lysis (left panel) and relative lysis (right panel) and the SEM of the three hiPSC lines by autologous NK cells is shown.
(PDF)

S4 Fig Human iPSC lines were used as target cells for freshly isolated or IL-2-activated NK cells of three allogeneic donors in ^51^Cr-release assays. NK cells of three different donors ((A) donor 4, (B) donor 5, (C) donor 7) were isolated and used as effectors at day 0 (d0, left panels) or after stimulation with IL-2 (200 U/ml) for 4 days (d4, right panels). The means of specific lysis and the SEM at different effector:target (E:T) ratios (16:1 to 0.25:1 for resting NK cells and 4:1 to 0.06:1 for IL2-activated NK cells) are shown to summarize these experiments. The reference target cell line K562 was included in every experiment in addition to the hiPSC lines D1-iPSC4, D2-iPSC1, and D6-iPSC2. Each individual test was done in triplicates. The numbers of individual experiments (n) are indicated in the figure.
(PDF)

S5 Fig Phenotypic characterization of NK cells. MACS-purified NK cells from three blood donors were analyzed by flow cytometry at day 0 (d0) and after stimulation for four days (d4) with IL-2 (200 U/ml). The percentages of cells positive for the indicated NK cell markers are shown as means plus SEM of three individual experiments. The CD56^dim^ and CD56^bright^ populations were not clearly distinguishable anymore at day 4 after stimulation with IL-2.
(PDF)

S6 Fig The KIR repertoire of NK cell donors was characterized by flow cytometry. The reactivity of a panel of anti-KIR mAbs against CD56^+^CD3^-^ NK cells of NK cell donors 4 (A), 5 (B) and 7 (C) was tested. The clone numbers and the reported reactivity against individual KIR molecules are indicated. KIR molecules, which could be present according to the KIR genotype of the donors (see S2 Table) are indicated by color. Inhibitory KIRs are marked in red and activating KIRs in green. Means and SEM of three experiments are shown.
(PDF)

S7 Fig NK cells degranulating in response to K562 cells are enriched for several NK cell receptors. In the left panel a summary of means and the SEM of NKG2D^+^, NKG2A^+^, DNAM-1^+^, and KIR^+^ cells among all NK cells of donors 4, 5, and 7 exposed to K562 as well as CD107a^-^ and CD107a^+^ NK cells is shown. In the right panel a summary of means and the SEM of the MFI of NKG2D, NKG2A, DNAM-1, and KIR on all NK cells exposed to K562 cells as well as CD107a^-^ and CD107a^+^ NK cells is shown. Significant differences between CD107a^-^ and CD107a^+^ NK cells are indicated (n = 26, ** P<0.01, * P<0.05, *t*-test after Bonferroni-Holm correction).
(PDF)

S8 Fig Enrichment of NK cells degranulating in response to hiPSC lines for specific NK cell receptors is more influenced by the NK cell donors than by the hiPSC lines. The difference between CD107a^-^ and CD107a^+^ NK cells was calculated for NKG2D^+^ (A), NGK2A^+^ (C), KIR^+^ NK cells (E) and for the expression intensities (MFI) of these molecules (B, D, F). The data are shown as means and SEM and they were grouped for the three donors (left panels) or the three hiPSC lines (right panels). Significant differences were calculated by 2-way-ANOVA adjusted either for the NK cell donors or the hiPSC lines and are indicated in the figure (n = 9).
(PDF)

S9 Fig The NK cell receptor repertoire of NK cells degranulating in response to hiPSC lines and K562 cells is shown for individual NK cell donors and hiPSC targets. The difference between CD107a^-^ and CD107a^+^ NK cells was calculated for NKG2D^+^ (A, left panel), NGK2A^+^ (B, left panel), DNAM-1^+^ (C, left panel), and KIR^+^ NK cells (D, left panel). The difference between CD107a^-^ and CD107a^+^ NK cells was also calculated for the expression intensities (MFI) of these molecules, i. e. NKG2D (A, right panel), NGK2A (B, right panel), DNAM-1 (C, right panel), and KIR (D, right panel). The data are shown as means and SEM of three individual experiments and they were grouped for the three donors (left panels) or the three hiPSC lines (right panels). Significant differences between the donors are indicated in the figure (n = 3, * P<0.05, *t*-test).
(PDF)

S10 Fig Inhibition of killing of hiPSC lines and K562 cells by mAbs is shown for individual NK cell donors and target cells. Means of specific lysis and SEM of three hiPSC lines and K562 cells by IL-2-activated NK cells from donor 4 (A), donor 5 (C), and donor 7 (E) in ^51^Cr-release assay is shown after incubation with an isotype control (IgG_1_) or blocking mAbs against NKG2D (aNKG2D), DNAM-1 (aDNAM-1), or ICAM-1 (aICAM-1) at a concentration of 10 μg/ml. Means of specific lysis and SEM of three hiPSC lines and K562 cells by IL-2-stimulated NK cells from donor 4 (B), donor 5 (D), and donor 7 (F) in ^51^Cr-release assays is shown after incubation with the W6/32 HL mAb that binds to HLA class I molecules or as control the non-binding variant W6/32 HK at a concentration of 10 μg/ml. Significant differences between the mAbs are indicated in the figure (n = 3, * P<0.05, *t*-test).
(PDF)

S1 Table Genes analyzed by qPCR and primers used. (PDF)

S2 Table KIR genotypes of NK cell donors 4, 5, and 7. (PDF)
