# Supplementary material for: Human Induced Pluripotent Stem Cells Are Targets for Allogeneic and Autologous Natural Killer (NK) Cells and Killing Is Partly Mediated by the Activating NK Receptor DNAM-1
Source: PLoS One. 2015 May 7;10(5):e0125544. doi: 10.1371/journal.pone.0125544 (PMC4423859; doi:10.1371/journal.pone.0125544)

S1 Fig. Human iPSC lines were used as target cells for purified and IL-2-activated NK cells of either various allogeneic or autologous donors in ^{51}Cr-release assays.

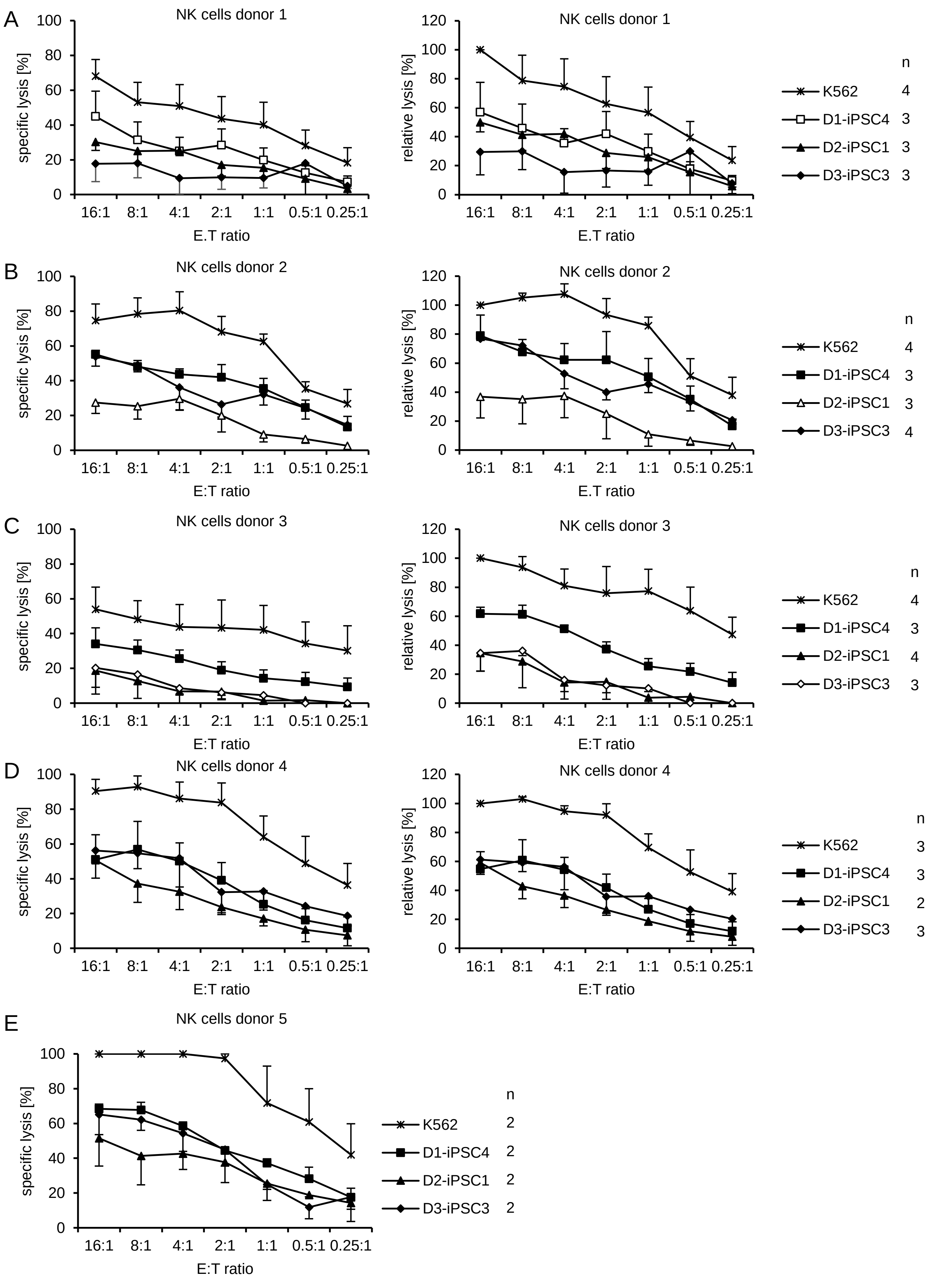

Supplement: S1 Fig — The reference target cell line K562 was included in every experiment. Each individual test was done in triplicates. The means of specific lysis and the standard error of the mean (SEM) at different effector:target (E:T) ratios (16:1 to 0.25:1) are shown to summarize these experiments (left panels). In addition, the killing of the reference K562 cells at the highest effector to target ratio (16:1) was set to 100% in each individual experiment and the relative lysis of the other target cell lines and at the various effector to target ratios was calculated accordingly (right panels). The relative lysis is not shown for NK cells of donor 5 since the specific lysis of K562 cells was 100% leading to an identity of specific and relative lysis. The results are grouped with respect to the NK cell donors, i.e. (A) donor 1, (B) donor 2, (C) donor 3, (D) donor 4, and (E) donor 5. In panels A, B, and C, the respective autologous hiPSC line is indicated by open symbols. Allogeneic hiPSC target cell lines are indicated by closed symbols. The numbers of individual experiments (n) are indicated in the figure. (PDF) [file pone.0125544.s001.pdf]
