# Supplementary material for: Human Induced Pluripotent Stem Cells Are Targets for Allogeneic and Autologous Natural Killer (NK) Cells and Killing Is Partly Mediated by the Activating NK Receptor DNAM-1
Source: PLoS One. 2015 May 7;10(5):e0125544. doi: 10.1371/journal.pone.0125544 (PMC4423859; doi:10.1371/journal.pone.0125544)

S2 Fig. Human iPSC lines were killed by purified and IL-2-activated NK cells of various donors but allogeneic effector cells were more efficient than autologous NK cells.

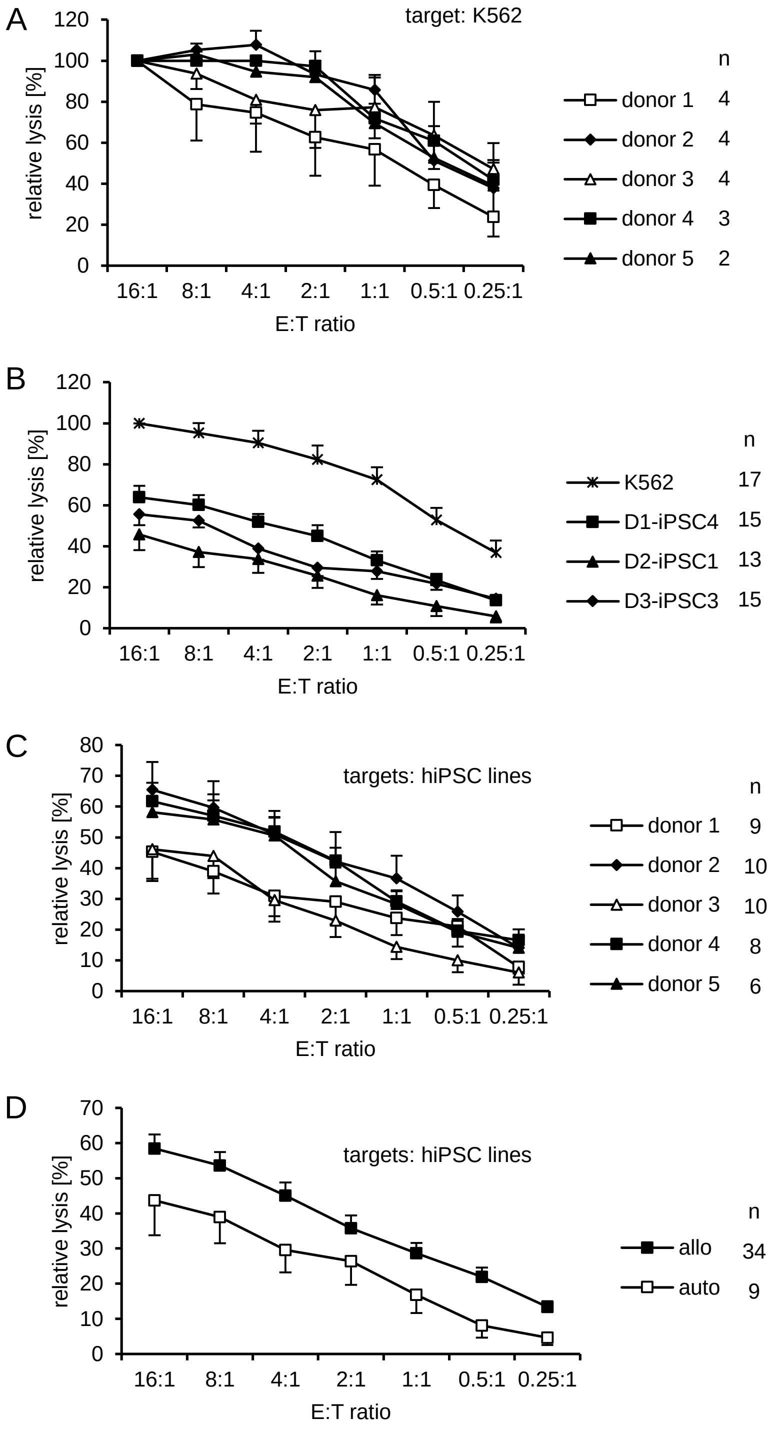

Supplement: S2 Fig — The same data set as in Fig 2 is shown but now the killing of K562 cells at the highest effector to target ratio (16:1) was set to 100% in each individual experiment and the relative lysis of the other target cell lines and at the various effector to target ratios was calculated accordingly. The numbers of individual experiments (n) are indicated in the figure. (A) NK cells from five donors were stimulated for four days with IL-2 (200 U/ml) and used as effector cells against the reference target cell line K562 in 51Cr-release assays. Each individual test was done in triplicates. The means of relative lysis and the SEM at E:T ratios 16:1 to 0.25:1 are shown to summarize these experiments. (B) A summary of means of relative lysis and the SEM of K562 and three hiPSC lines by IL-2-activated NK cells from five donors (1 to 5) is shown. (C) A summary of means of relative lysis and the SEM of the three hiPSC lines (D1-iPSC4, D2-iPSC1, D3-iPSC3) by IL-2-activated NK cells of five different donors is shown. (D) A summary of means of relative lysis and the SEM of the three hiPSC lines (D1-iPSC4, D2-iPSC1, D3-iPSC3) by IL-2-activated allogeneic (allo) and autologous (auto) NK cells is shown. (PDF) [file pone.0125544.s002.pdf]
