# Supplementary material for: Human Induced Pluripotent Stem Cells Are Targets for Allogeneic and Autologous Natural Killer (NK) Cells and Killing Is Partly Mediated by the Activating NK Receptor DNAM-1
Source: PLoS One. 2015 May 7;10(5):e0125544. doi: 10.1371/journal.pone.0125544 (PMC4423859; doi:10.1371/journal.pone.0125544)

S3 Fig. Human iPSC lines were killed by purified and IL-2-activated allogeneic or autologous NK cells of various donors but with different efficacy.

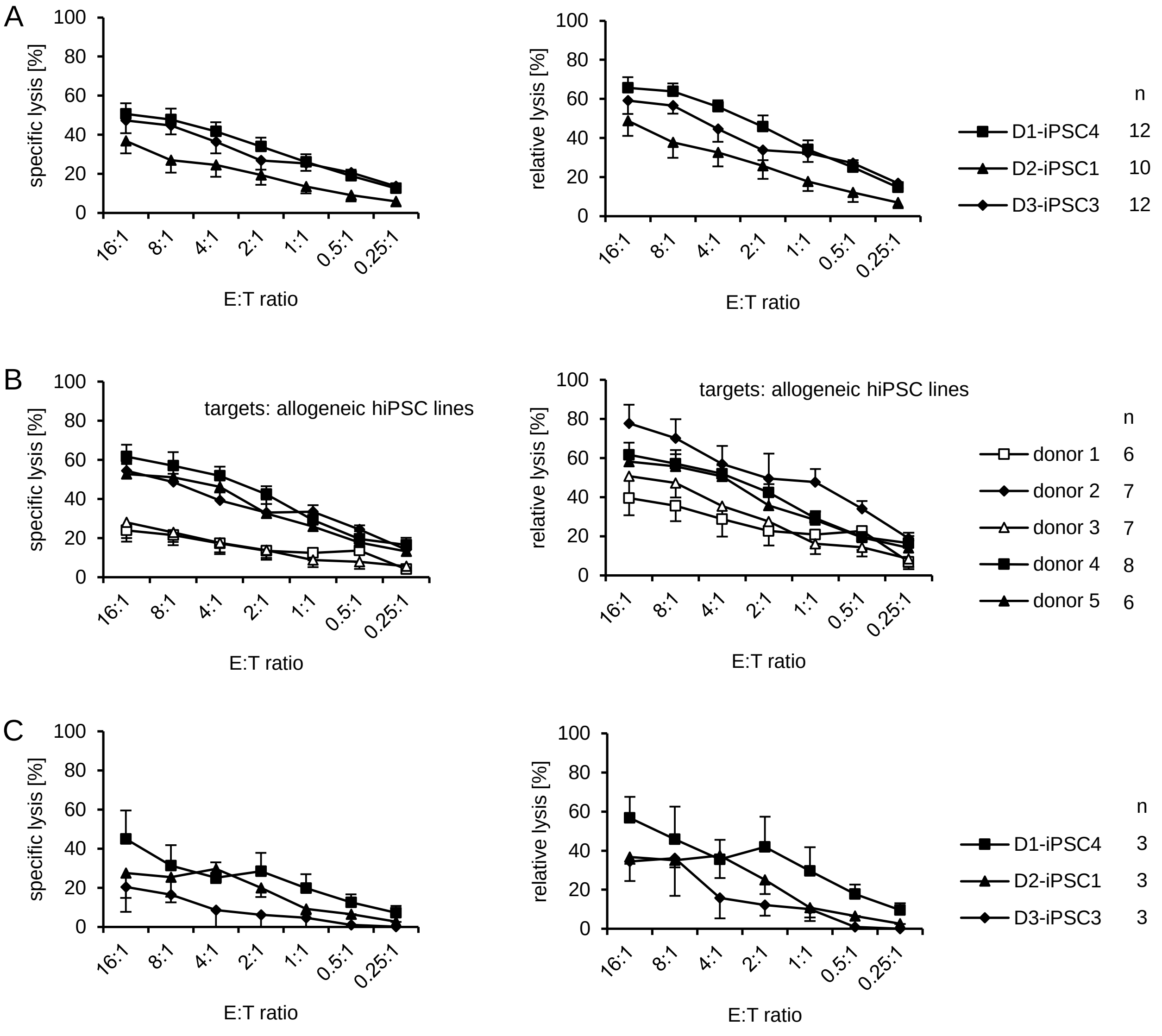

Supplement: S3 Fig — (A) A summary of means of specific lysis (left panels) and relative lysis (adjusted to killing of K562 cells, right panels) and the SEM of three hiPSC lines by allogeneic IL-2-activated NK cells from four donors (donors 1 to 5) is shown. The numbers of individual experiments (n) are indicated in the figure. (B) A summary of means of specific lysis (left panel) and relative lysis (right panel) and the SEM of allogeneic hiPSC lines (D1-iPSC4, D2-iPSC1, D3-iPSC3) by NK cells of five different donors is shown. (C) A summary of means of specific lysis (left panel) and relative lysis (right panel) and the SEM of the three hiPSC lines by autologous NK cells is shown. (PDF) [file pone.0125544.s003.pdf]
