# Supplementary material for: Human Induced Pluripotent Stem Cells Are Targets for Allogeneic and Autologous Natural Killer (NK) Cells and Killing Is Partly Mediated by the Activating NK Receptor DNAM-1
Source: PLoS One. 2015 May 7;10(5):e0125544. doi: 10.1371/journal.pone.0125544 (PMC4423859; doi:10.1371/journal.pone.0125544)

S4 Fig. Human iPSC lines were used as target cells for freshly isolated or IL-2-activated NK cells of three allogeneic donors in ^{51}Cr-release assays.

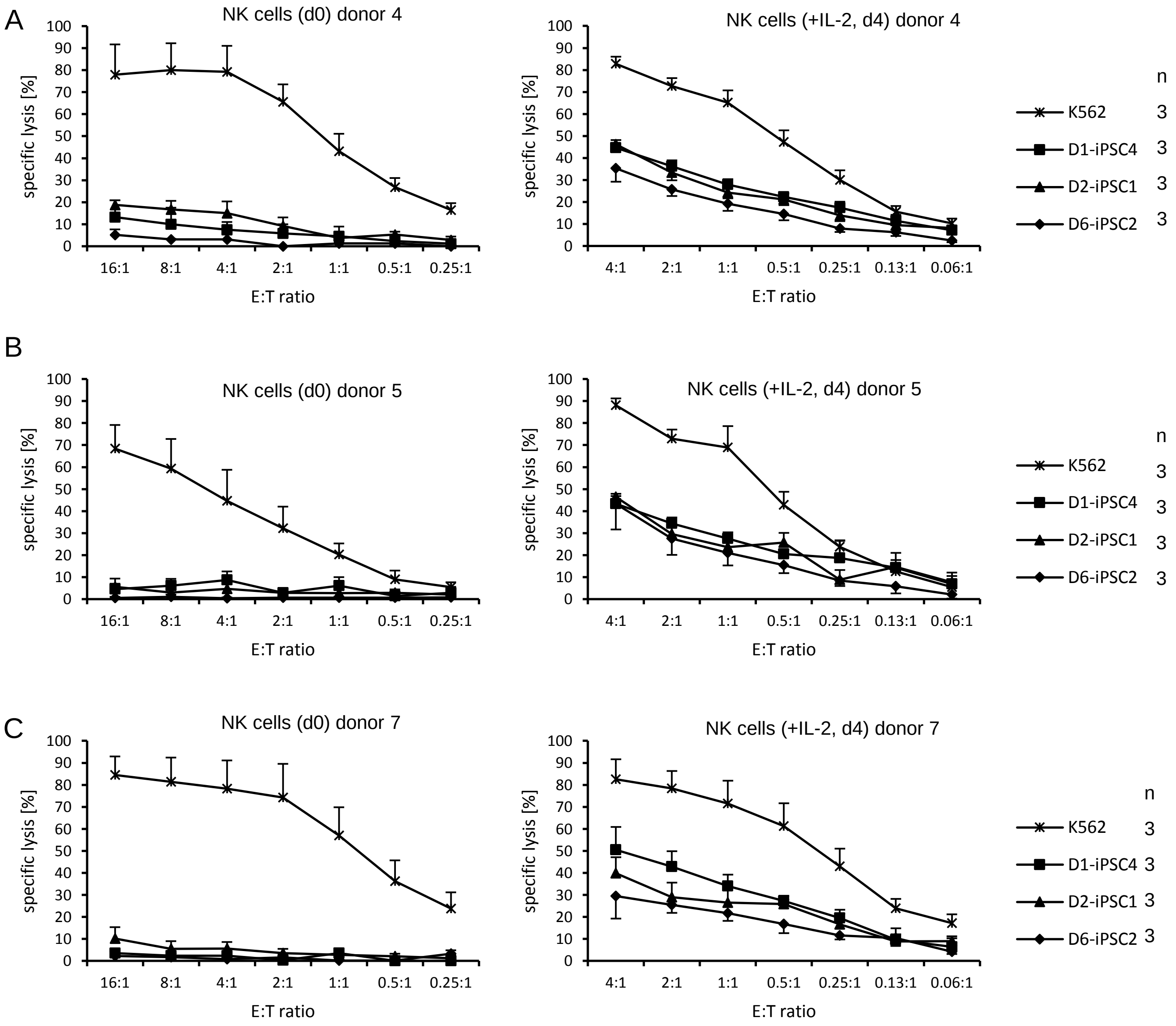

Supplement: S4 Fig — NK cells of three different donors ((A) donor 4, (B) donor 5, (C) donor 7) were isolated and used as effectors at day 0 (d0, left panels) or after stimulation with IL-2 (200 U/ml) for 4 days (d4, right panels). The means of specific lysis and the SEM at different effector:target (E:T) ratios (16:1 to 0.25:1 for resting NK cells and 4:1 to 0.06:1 for IL2-activated NK cells) are shown to summarize these experiments. The reference target cell line K562 was included in every experiment in addition to the hiPSC lines D1-iPSC4, D2-iPSC1, and D6-iPSC2. Each individual test was done in triplicates. The numbers of individual experiments (n) are indicated in the figure. (PDF) [file pone.0125544.s004.pdf]
