# Supplementary figures and images for: Human Induced Pluripotent Stem Cells Are Targets for Allogeneic and Autologous Natural Killer (NK) Cells and Killing Is Partly Mediated by the Activating NK Receptor DNAM-1
Source: PLoS One. 2015 May 7;10(5):e0125544. doi: 10.1371/journal.pone.0125544 (PMC4423859; doi:10.1371/journal.pone.0125544)

**S5 Fig.** Phenotypic characterization of NK cells.

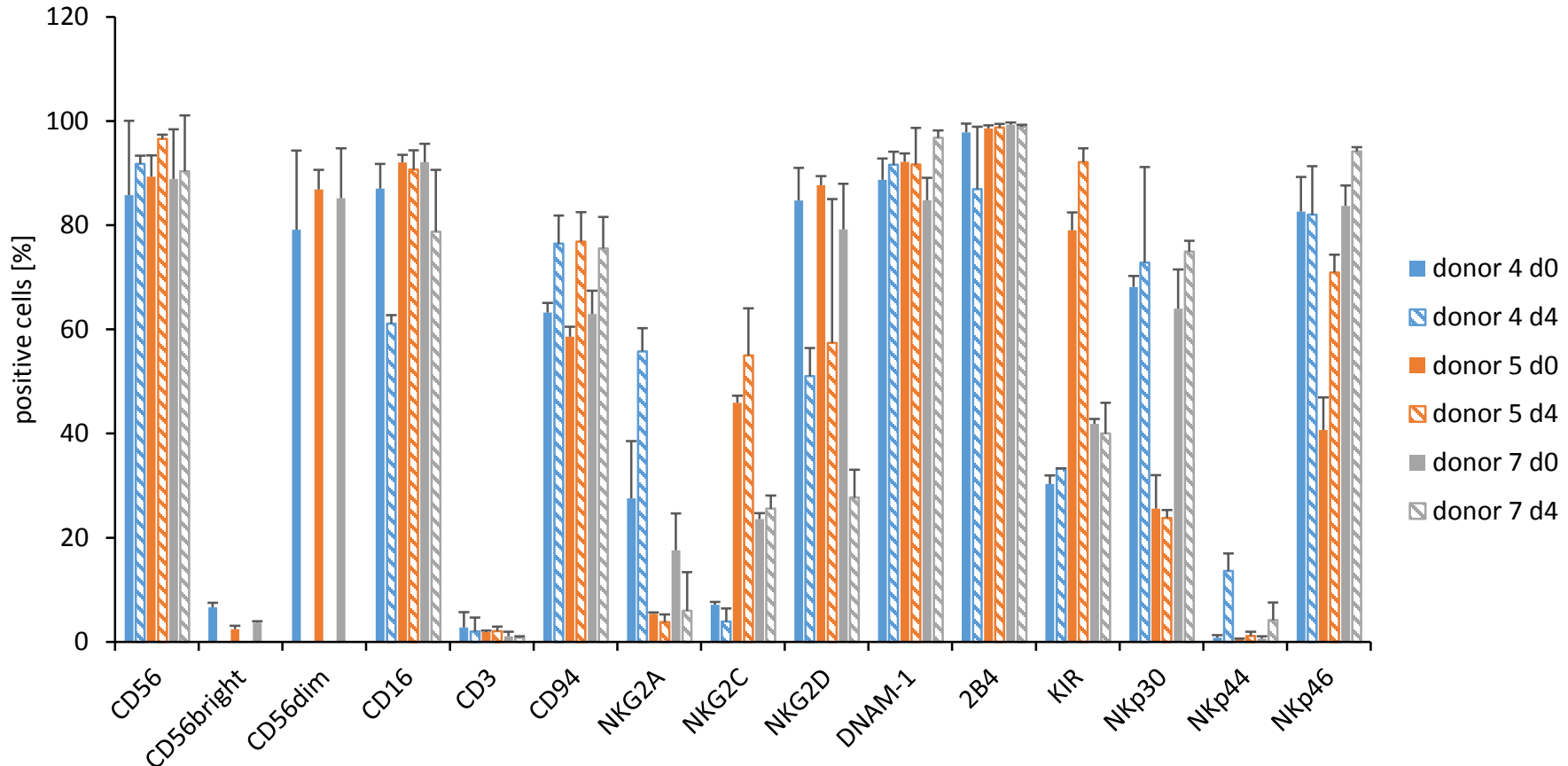

Supplement: S5 Fig — MACS-purified NK cells from three blood donors were analyzed by flow cytometry at day 0 (d0) and after stimulation for four days (d4) with IL-2 (200 U/ml). The percentages of cells positive for the indicated NK cell markers are shown as means plus SEM of three individual experiments. The CD56dim and CD56bright populations were not clearly distinguishable anymore at day 4 after stimulation with IL-2. (PDF) [file pone.0125544.s005.pdf]

**S6 Fig.** The KIR repertoire of NK cell donors was characterized by flow cytometry.

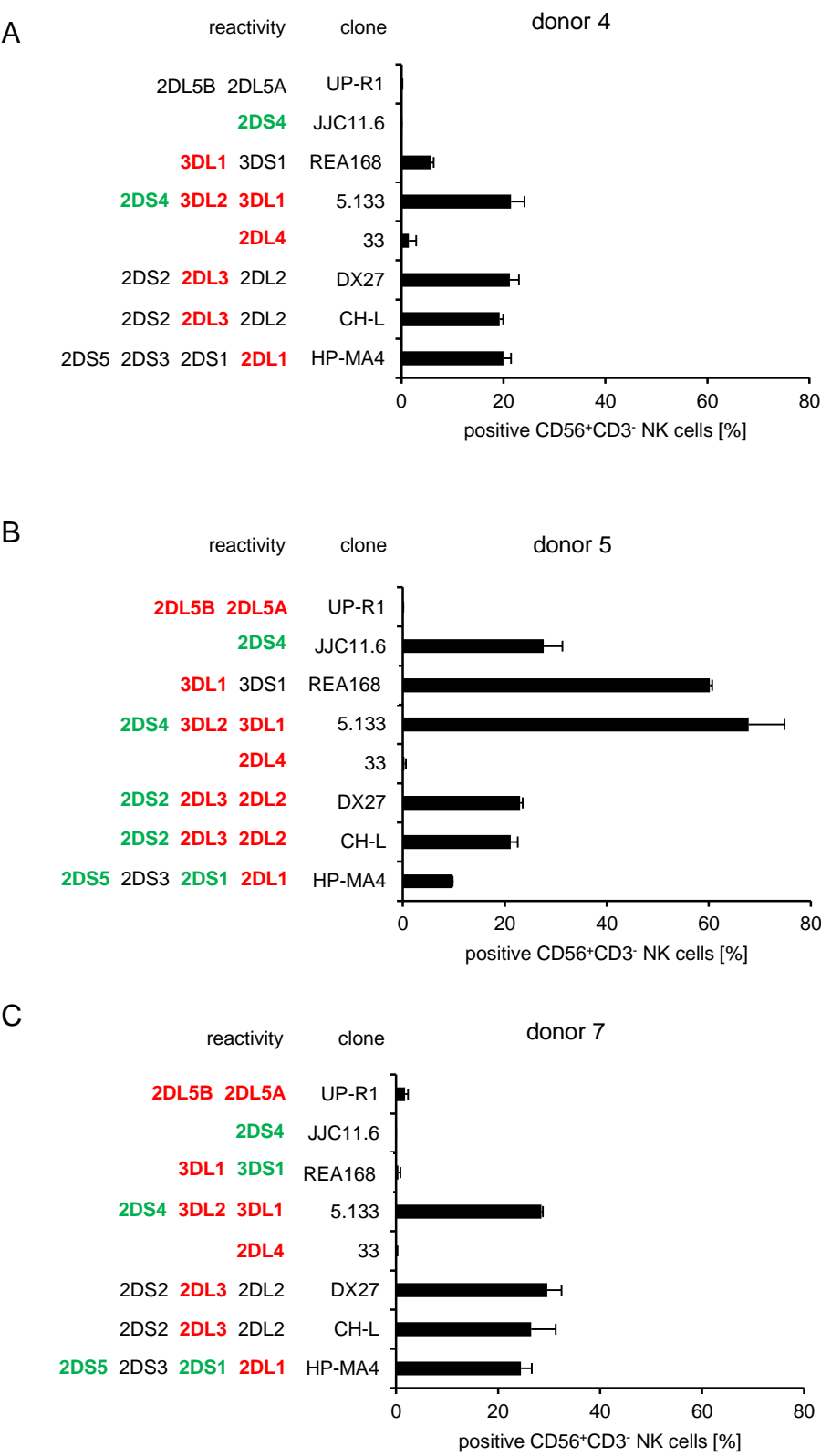

Supplement: S6 Fig — The reactivity of a panel of anti-KIR mAbs against CD56+CD3- NK cells of NK cell donors 4 (A), 5 (B) and 7 (C) was tested. The clone numbers and the reported reactivity against individual KIR molecules are indicated. KIR molecules, which could be present according to the KIR genotype of the donors (see S2 Table) are indicated by color. Inhibitory KIRs are marked in red and activating KIRs in green. Means and SEM of three experiments are shown. (PDF) [file pone.0125544.s006.pdf]

**S7 Fig.** NK cells degranulating in response to K562 cells are enriched for several NK cell receptors.

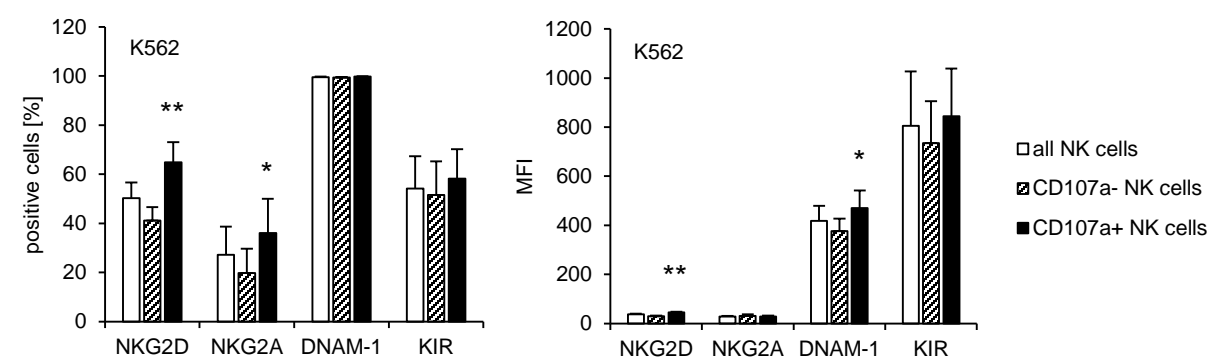

Supplement: S7 Fig — In the left panel a summary of means and the SEM of NKG2D+, NKG2A+, DNAM-1+, and KIR+ cells among all NK cells of donors 4, 5, and 7 exposed to K562 as well as CD107a- and CD107a+ NK cells is shown. In the right panel a summary of means and the SEM of the MFI of NKG2D, NKG2A, DNAM-1, and KIR on all NK cells exposed to K562 cells as well as CD107a- and CD107a+ NK cells is shown. Significant differences between CD107a- and CD107a+ NK cells are indicated (n = 26, ** P<0.01, * P<0.05, t-test after Bonferroni-Holm correction). (PDF) [file pone.0125544.s007.pdf]
