# Supplementary material for: Human Induced Pluripotent Stem Cells Are Targets for Allogeneic and Autologous Natural Killer (NK) Cells and Killing Is Partly Mediated by the Activating NK Receptor DNAM-1
Source: PLoS One. 2015 May 7;10(5):e0125544. doi: 10.1371/journal.pone.0125544 (PMC4423859; doi:10.1371/journal.pone.0125544)

S8 Fig. Enrichment of NK cells degranulating in response to hiPSC lines for specific NK cell receptors is more influenced by the NK cell donors than by the hiPSC lines.

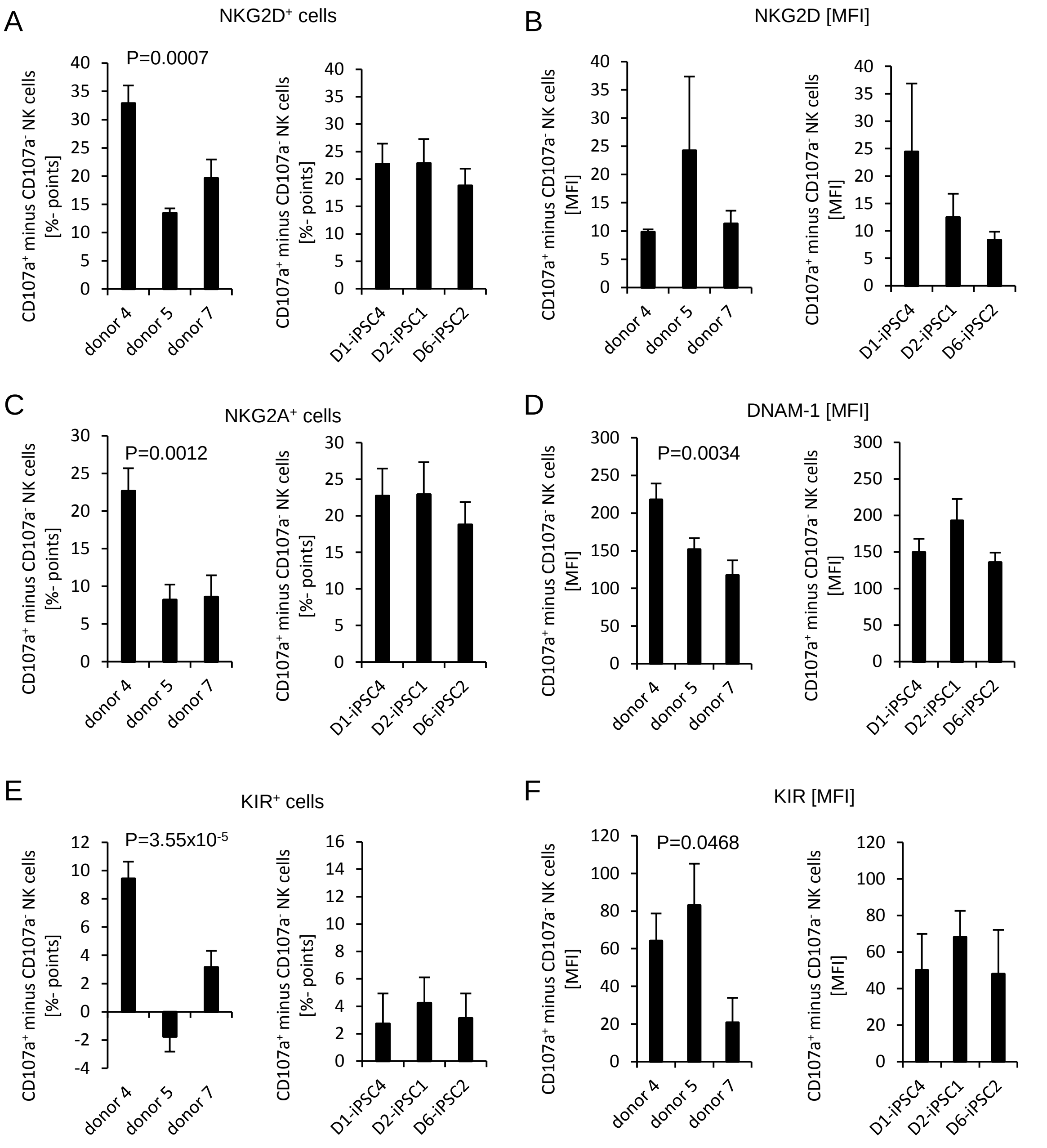

Supplement: S8 Fig — The difference between CD107a- and CD107a+ NK cells was calculated for NKG2D+ (A), NGK2A+ (C), KIR+ NK cells (E) and for the expression intensities (MFI) of these molecules (B, D, F). The data are shown as means and SEM and they were grouped for the three donors (left panels) or the three hiPSC lines (right panels). Significant differences were calculated by 2-way-ANOVA adjusted either for the NK cell donors or the hiPSC lines and are indicated in the figure (n = 9). (PDF) [file pone.0125544.s008.pdf]
