# Supplementary material for: Human Induced Pluripotent Stem Cells Are Targets for Allogeneic and Autologous Natural Killer (NK) Cells and Killing Is Partly Mediated by the Activating NK Receptor DNAM-1
Source: PLoS One. 2015 May 7;10(5):e0125544. doi: 10.1371/journal.pone.0125544 (PMC4423859; doi:10.1371/journal.pone.0125544)

**S9 Fig.** The NK cell receptor repertoire of NK cells degranulating in response to hiPSC lines and K562 cells is shown for individual NK cell donors and hiPSC targets.

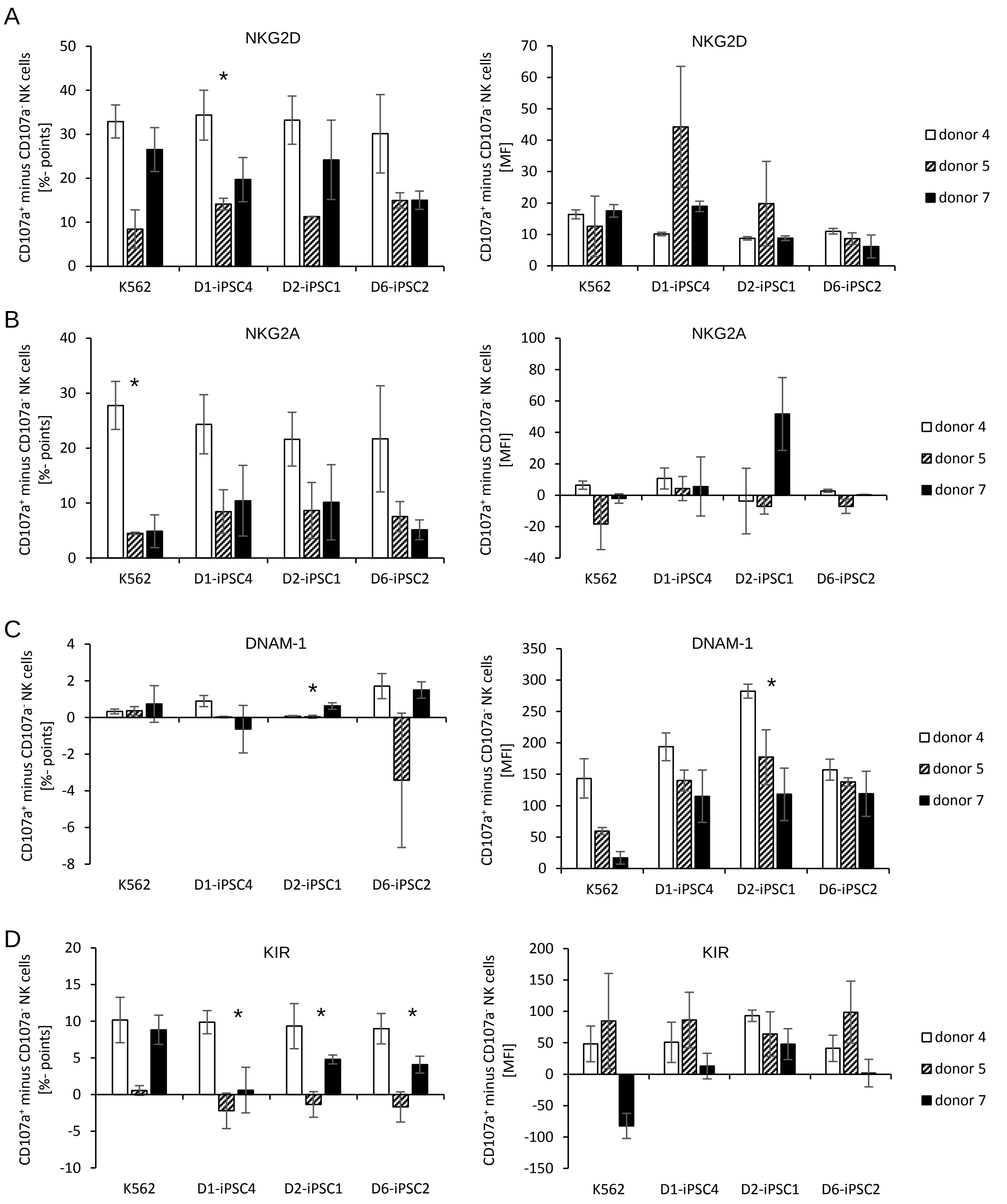

Supplement: S9 Fig — The difference between CD107a- and CD107a+ NK cells was calculated for NKG2D+ (A, left panel), NGK2A+ (B, left panel), DNAM-1+ (C, left panel), and KIR+ NK cells (D, left panel). The difference between CD107a- and CD107a+ NK cells was also calculated for the expression intensities (MFI) of these molecules, i. e. NKG2D (A, right panel), NGK2A (B, right panel), DNAM-1 (C, right panel), and KIR (D, right panel). The data are shown as means and SEM of three individual experiments and they were grouped for the three donors (left panels) or the three hiPSC lines (right panels). Significant differences between the donors are indicated in the figure (n = 3, * P<0.05, t-test). (PDF) [file pone.0125544.s009.pdf]
