# Supplementary material for: Human Induced Pluripotent Stem Cells Are Targets for Allogeneic and Autologous Natural Killer (NK) Cells and Killing Is Partly Mediated by the Activating NK Receptor DNAM-1
Source: PLoS One. 2015 May 7;10(5):e0125544. doi: 10.1371/journal.pone.0125544 (PMC4423859; doi:10.1371/journal.pone.0125544)

S10 Fig. Inhibition of killing of hiPSC lines and K562 cells by mAbs is shown for individual NK cell donors and target cells.

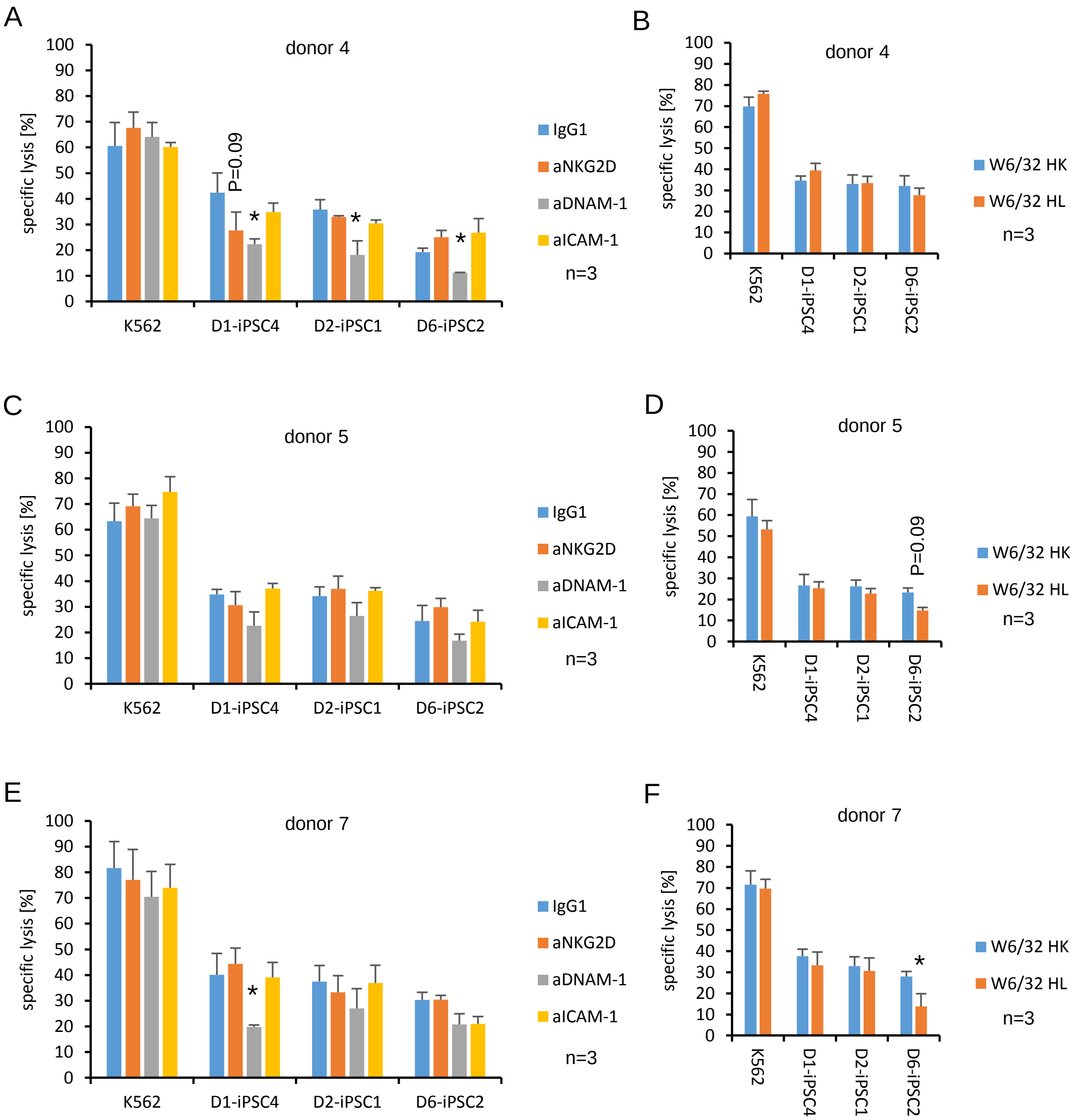

Supplement: S10 Fig — Means of specific lysis and SEM of three hiPSC lines and K562 cells by IL-2-activated NK cells from donor 4 (A), donor 5 (C), and donor 7 (E) in 51Cr-release assay is shown after incubation with an isotype control (IgG1) or blocking mAbs against NKG2D (aNKG2D), DNAM-1 (aDNAM-1), or ICAM-1 (aICAM-1) at a concentration of 10 μg/ml. Means of specific lysis and SEM of three hiPSC lines and K562 cells by IL-2-stimulated NK cells from donor 4 (B), donor 5 (D), and donor 7 (F) in 51Cr-release assays is shown after incubation with the W6/32 HL mAb that binds to HLA class I molecules or as control the non-binding variant W6/32 HK at a concentration of 10 μg/ml. Significant differences between the mAbs are indicated in the figure (n = 3, * P<0.05, t-test). (PDF) [file pone.0125544.s010.pdf]
