# Supplementary material for: Human Induced Pluripotent Stem Cells Are Targets for Allogeneic and Autologous Natural Killer (NK) Cells and Killing Is Partly Mediated by the Activating NK Receptor DNAM-1
Source: PLoS One. 2015 May 7;10(5):e0125544. doi: 10.1371/journal.pone.0125544 (PMC4423859; doi:10.1371/journal.pone.0125544)
Supplement: S1 Table — (PDF) [file pone.0125544.s011.pdf]

S1 Table. Genes analyzed by qPCR and primers used.

Gene			Primer				Product	
Abbreviation	Name(s)	NCBI/Database	Start position	Length[bp]	Sequence<br>Forward<br>Reverse	Tm[1]<br>[°C]	Size<br>[bp]	Tm<br>[°C]
ACTB	β-actin	NM_001101.3	85	24	ATGGATGATGATATCGCCGCGCTC	61.0	161	87.5
			245	26	ACATAGGAATCCTTCTGACCCATGCC	60.0		
B2M	β2-microglobulin	NM_004048.2	203	21	CTGGGTTTCATCCATCCGACA	56.8	166	80.6
			368	23	TTCACACGGCAGGCATACTCATC	59.1		
CANX	Calnexin	NM_001746.3	181	24	GAAGGGAAGTGGTTGCTGTGTATG	57.7	156	80.6
			336	24	AGGAGGAGCAGTGGTATCTGGTTT	59.4		
CALR	Calreticulin or Calregulin	NM_004343.3	837	23	TGGGATGAAGAGATGGACGGAGA	59.0	164	82.2
			1000	24	GCATAGATACTGGGATCGGGAGAA	57.5		
CD48	Cluster of differentiation-48; B lymphocyte activation marker BLAST-1 (*BCM1*)	NM_001778.3	235	25	GCCTGAGAACTACAAACAACTAACC	55.6	230	81.5
			464	25	GCAGCTTGATCTTCCATTCTTGCTC	58.1		
CD112	Cluster of differentiation-112 or HS poliovirus receptor-related 2 (*PVRL2*)	NM_002856.2	989	21	TGGACTGGGAAGCCAAAGAGA	58.0	173	85.6
			1161	25	TACAGAGAGGGTCACAGGTATCAGG	58.7		
CD155	Cluster of differentiation-155 or Homo sapiens poliovirus receptor (*PVR*)	NM_001135769.1	5454	24	GCTCTGCTGTTTGTTCTGCTTTCC	57.0	165	85.0
			5618	22	TTTCTGCTGCTGGATGCGGTTT	57.9		
DRA	Major histocompatibility complex, class II, DR alpha, HLA-DRA	NM_019111.4	616	24	TTTCCGCAAGTTCCACTATCTCCC	58.6	193	85.0
			808	24	AATAATGATGCCCACCAGACCCAC	59.1		
ERp57	Endoplasmic reticulum resident protein 57 or *PDIA3*, ERp60/61	NM_005313.4	1502	24	ACCATATACTTCTCTCCAGCCAAC	55.9	162	79.5
			1663	23	GAGATCCTCCTGTGCCTTCTTCT	57.8		
GAPDH	Glycerinaldehyd-3-phosphat-dehydrogenase	NM_002046.3	1121	24	ACGAATTTGGCTACAGCAACAGGG	59.6	188	84.9
			1308	23	TCTACATGGCAACTGTGAGGAGG	58.2		

Gene			Primer				Product	
Abbreviation	Name	NCBI/Database	Start position	Length[bp]	Sequence Forward Reverse	Tm[1] [°C]	Size [bp]	Tm [°C]
HLA-A	Human leucocyte antigen A	www.ebi.ac.uk	186	17	CGACGCCGCGAGCCAGA	63.7	261	88.3
			446	24	GCGATGTAATCCTTGCCGTCGTAG	59.4		
HLA-B	Human leucocyte antigen B	www.ebi.ac.uk	427	26	GACGGCAAGGATTACATCGCCCTGAA	62.4	100	85.8
			526	18	CACGGGCCGCCTCCCACT	65.8		
HLA-C	Human leucocyte antigen C	www.ebi.ac.uk	258	21	GGAGACACAGAAGTACAAGCG	55.1	173	87.3
			430	22	CGTCGTAGGCGTACTGGTCATA	57.8		
HLA-E	Human leucocyte antigen E	www.ebi.ac.uk	413	16	CCTACGACGGCAAGGA	53.7	175	85.8
			587	20	CCCTTCTCCAGGTATTTGTG	52.5		
HLA-F	Human leucocyte antigen F	www.ebi.ac.uk	631	24	GGCAGAGGAATATGCAGAGGAGTT	58.3	243	87.5
			873	19	TCTGTGTCCTGGGTCTGTT	55.3		
HLA-G	Human leucocyte antigen G	www.ebi.ac.uk	249	23	TTGGGAAGAGGAGACACGGAACA	59.7	132	84.2
			380	21	AGGTCGCAGCCAATCATCCAC	59.8		
HPRT	Hypoxanthine-guanine phosphoribosyl-transferase	NM_000194.2	613	24	TGGTCAGGCAGTATAATCCAAAGA	55.5	137	79.8
			749	25	GTCAAGGGCATATCCTACAACAAAC	55.7		
ICAM1	Intercellular adhesion molecule 1 or *CD54*	NM_000201.2	2135	24	GACTAAGCCAAGAGGAAGGAGCAA	58.4	154	81.6
			2288	24	TCAGCATACCCAATAGGCAGCAAG	59		
LMP2	Large multifunctional protease 2; low molecular weight protein 2; *PSMB9*	NM_002800.4	168	23	GATGGGTTCTGATTCCCGAGTGT	58.5	181	86.8
			348	21	AGGTTCCTCCAGTTCTATCCC	55.4		
LMP7	Large multifunctional protease 7; low molecular weight protein 7; *PSMB8*	NM_148919.3	346	22	ACGGGTGAACAAGGTGATTGAG	58.2	218	85.8
			563	23	AGATCATACTGCCCATAGAGAGG	55.1		

Gene			Primer				Product	
Abbreviation	Name	NCBI/Database	Start position	Length[bp]	Sequence<br>Forward<br>Reverse	Tm[1]<br>[°C]	Size<br>[bp]	Tm<br>[°C]
MICA	MHC class I polypeptide-related sequence A	www.ebi.ac.uk	263	22	ACTTGACAGGGAACGGAAAGGA	58	148	82.5
			410	23	CCATCGTAGTAGAAATGCTGGGA	56.1		
MICB	MHC class I polypeptide-related sequence B	www.ebi.ac.uk	117	22	ATCTGTGCAGTCAGGGTTTCTC	56.7	173	85.4
			289	22	TGAGGTCTTGCCCATTCTCTGT	57.9		
TAP1	Transporter associated with antigen processing 1	NM_000593.5	910	23	TCCTGGTGGTCCTCTCCTCTCTT	60.6	181	83.4
			1090	24	GTGTTGTTATAGATCCCGTCACCC	56.9		
TAP2	Transporter associated with antigen processing 2	NM_018833.2	934	23	TCTTGCGAAGCCTGGTGAAAGTG	59.6	139	84.5
			1072	22	ACTTCCTGATGGCGGGTGTTGT	61		
TAPBP	TAP-binding protein or Tapasin	NM_172208.2	1541	22	ACCCTGGAGGTAGCAGGTCTTT	59.7	133	84.6
			1673	23	AATCCTTGCAGGTGGACAGGTAG	58.6		
ULBP1	UL16 binding protein 1 or NKG2DL1; *RAET1L*	NM_025218.2	2790	24	TGGGTATCATGCTTACTGTCTGGG	58.1	211	79.6
			3000	25	GGGTTTGGGTTCATAGTGCAGAGTT	59		
ULBP2	UL16 binding protein 2 or NKG2DL2; *RAET1H*	NM_025217.2	764	22	CTTTGCTGCCTCCTCATCATCC	57.8	138	83.2
			901	21	GCCAGACAGAAGGGCGAGTTT	59.6		

[1]Tm: melting temperature.
