# Supplementary material for: Human Induced Pluripotent Stem Cells Are Targets for Allogeneic and Autologous Natural Killer (NK) Cells and Killing Is Partly Mediated by the Activating NK Receptor DNAM-1
Source: PLoS One. 2015 May 7;10(5):e0125544. doi: 10.1371/journal.pone.0125544 (PMC4423859; doi:10.1371/journal.pone.0125544)
Supplement: S2 Table — (PDF) [file pone.0125544.s012.pdf]

S2 Table. KIR genotypes of NK cell donors 4, 5, and 7.

	donor 4	donor 5	donor 7
**KIR2DL1**	+	+	+
**KIR2DL2**	-	+	-
**KIR2DL3**	+	+	+
**KIR2DL4**	+	+	+
**KIR2DL5**	-	+	+
**KIR3DL1**	+	+	+
**KIR3DL2**	+	+	+
**KIR3DL3**	+	+	+
**KIR2DS1**	-	+	+
**KIR2DS2**	-	+	-
**KIR2DS3**	-	-	-
**KIR2DS4**	+	+	+
**KIR2DS5**	-	+	+
**KIR3DS1**	-	nd[1]	+
**Summary**	AA KIR	AB KIR	AB KIR[2]

[1]nd: not determined

[2]presumably one A and one cenA/telB haplotype is present in donor 7.
